# ﻿New species and records of Botryosphaeriales (Dothideomycetes) associated with tree dieback in Beijing, China

**DOI:** 10.3897/mycokeys.106.122890

**Published:** 2024-06-27

**Authors:** Yingying Wu, Cheng Peng, Rong Yuan, Mingwei Zhang, Yang Hu, Chengming Tian

**Affiliations:** 1 The Key Laboratory for Silviculture and Conservation of the Ministry of Education, Beijing Forestry University, Beijing 100083, China Beijing Forestry University Beijing China; 2 The Forestry Protection Station of Tonghzou Strict in Beijing, Beijing 101100, China The Forestry Protection Station of Tonghzou Strict in Beijing Beijing China

**Keywords:** *Dothiorella*, morphology, *Phaeobotryon*, phylogeny, taxonomy

## Abstract

Botryosphaeriales species are important pathogens that have worldwide distribution. In this study, 23 Botryosphaeriales strains were isolated from 13 host species during a dieback disease survey in Beijing, China. Based on morphological and phylogenetic analyses, six Botryosphaeriales species were identified, including two new species named *Dothiorellahortiarborum***sp. nov.** and *Phaeobotryonfraxini***sp. nov.**, and four new host records: *Aplosporellaginkgonis* from Cotinuscoggygriavar.cinereus, *A.javeedii* from *Acermiyabei*, *Acertruncatum*, *Forsythiasuspensa*, *Lagerstroemiaindica*, *Sambucuswilliamsii*, *Syringavulgaris*, *Ulmuspumila*, *Xanthocerassorbifolium*, *A.yanqingensis* from *Acertruncatum*, and *Do.acericola* from *Forsythiasuspensa*, *Ginkgobiloba*, and *Syringaoblata*. This study enriches the species diversity associated with tree dieback in Beijing, China, and contributes to the further study of the taxonomy of this order.

## ﻿Introduction

Botryosphaeriales species are important plant pathogens commonly found on the trunks and branches of woody plants (Phillips et al. 2013; Lawrence et al. 2017; Zhu et al. 2018; Zhang et al. 2021). They are associated with branch canker, dieback, decline, and death, with consequences for the ecological and economic value of the forest (Slippers and Wingfield 2007; Phillips et al. 2013; Mohali-Castillo 2023). Botryosphaeriales species occur on a wide range of hosts, in the form of endophytes on woody plants and herbs, lichens, and even seaweed leaves in marine environments, suggesting that they have great potential for research value (Yang et al. 2017; Akinsanmi et al. 2019; Zhang et al. 2021; Mohali-Castillo 2023; Rathnayaka et al. 2023).

Phylogenetic analyses of DNA sequence data have an enormous influence on the systematics and taxonomy of the order Botryosphaeriales, including redefining families and genera and identifying new species (Phillips et al. 2019; Mohali-Castillo 2023). Schoch et al. (2006) combined SSU, LSU, *tef1-α*, and *rpb2* to first propose the order Botryosphaeriales, which contains only a single family of Botryosphaeriaceae. Minnis et al. (2012) supplemented the DNA sequence data of Planistromellaceae with phylogenetic analyses combining SSU, ITS, LSU, and *rpb1*, which introduced the family into the Botryosphaeriales. Wikee et al. (2013) reintroduced the Phyllostictaceae, grouped under Botryosphaeriales, to accommodate *Phyllosticta* using intronic genes (ITS, *act*, and *tef1-α*) and highly conserved coding regions of genes (LSU and GPDH). Slippers et al. (2013) added three new families, Aplosporellaceae (*Aplosporella* and *Bagnisiella*), Melanopsaceae (*Melanops*), and Saccharataceae (*Saccharata*), to Botryosphaeriales based on DNA sequence data of six loci (SSU, LSU, ITS, *tef1-α*, *tub2*, and mtSSU). Wyka and Broders (2016) introduced Septorioideaceae based on morphological and molecular evidence. Yang et al. (2017) mentioned that the LSU-*rpb2* combination could effectively classify taxa at the family and genus levels, and *rpb2* in combination with ITS, *tef1-α*, and *tub2* added additional resolution for species delimitation. For this reason, they combined the five fragments ITS, *tef1-α*, *tub2*, LSU, and *rpb2* to propose two new families, Endomelanconiopsisaceae and Pseudofusicoccumaceae. Therefore, Botryosphaeriales contained a total of nine families. However, Phillips et al. (2019) reassessed the families of Botryosphaeriales in terms of morphology of the sexual morphs and phylogenetic relationships of ITS and LSU sequence data, ultimately concluding that the order contained only six families (Aplosporellaceae, Botryosphaeriaceae, Melanopsaceae, Phyllostictaceae, Planistromellaceae, and Saccharataceae), with Endomelanconiopsisaceae, Pseudofusicoccumaceae, and Septorioideaceae as synonyms of existing families. Up to date, six families and 32 genera are accepted in Botryosphaeriales (https://www.outlineoffungi.org/). Of these, Botryosphaeriaceae is rich in species diversity, high in pathogenicity, and widely distributed.

Botryosphaeriaceae was first established by Theissen and Sydow (1918), containing three genera: *Botryosphaeria*, *Dibotryon*, and *Phaeobotryon*. Morphologically, Botryosphaericeae species are distinctive from other families in Botryosphaeriales by their large, ovoid to oblong, usually hyaline, aseptate ascospores (Phillips et al. 2013). Liu et al. (2012) assumed that ascospores could become pigmented and septate with age. Conidia in the asexual state of Botryosphaericeae are diverse in morphological characteristics (Phillips et al. 2005). Phylogenetically, however, there is a random distribution of hyaline or colored conidia or ascospores in the phylogenetic tree of Botryosphaericeae (Slippers et al. 2013). Therefore, accurate identification of species in the family by a single circumscription is not suitable. Currently, 22 genera and more than 200 species are contained within the family (https://www.outlineoffungi.org/). Recently, many new species have been introduced in the Botryosphaeriaceae, especially in the genera *Dothiorella* and *Phaeobotryon* (Jia et al. 2023; Li et al. 2023; Lin et al. 2023a; Wu et al. 2023).

Saccardo (1880) first established *Dothiorella* and designated *Do.pyrenophora* as the type species. Up to now, some scholars have made systematic revisions of *Dothiorella* to establish a more stable phylogenetic relationship (Dissanayake et al. 2016; Dissanayake et al. 2020; Zhang et al. 2021). The distinctive features of the genera are that the conidia are colored in the early stages of development, and with 1-septate, the sexual form of ascospores is brown and septate (Senanayake et al. 2023). The type species of the genus *Phaeobotryon* is *P.cercidis*, which is characterized by 2-septate brown ascospores with conical apiculate-elliptic to oblong or obovoid shapes at both ends and hyaline or brown conidia (Phillips et al. 2013; Fan et al. 2015b; Zhu et al. 2018; Pan et al. 2019).

In recent years, multiple studies have revealed that new species of Botryosphaeriales infest branches and trunks. Pan et al. (2019) found that *Phaeobotryonrhois* and *Diplodiaquercicola* were detrimental to *Rhustyphina* and *Quercusvariabilis* separately in Yudu Mountain, Beijing. *Aplosporellayanqingensis* and *Dothiorellabaihuashan* are mainly recorded on Pinaceae or Cupressaceae (Lin et al. 2023a). *Lasiodiplodiaregiae* caused the canker and dieback of apple trees (Wang et al. 2023). These studies suggest that Botryosphaeriales is rich in species diversity and has the potential to continue to be explored for new species. During the investigation of plant pathogens in Beijing, a higher number of diseased plant branches caused by Botryosphaeriales fungi were found. This study used phylogenetic analysis and morphological comparisons to describe new species and new host records, enriching the fungal taxa within Botryosphaeriales.

## ﻿Materials and method

### ﻿Sample collection and fungal isolation

**Figure 1.**
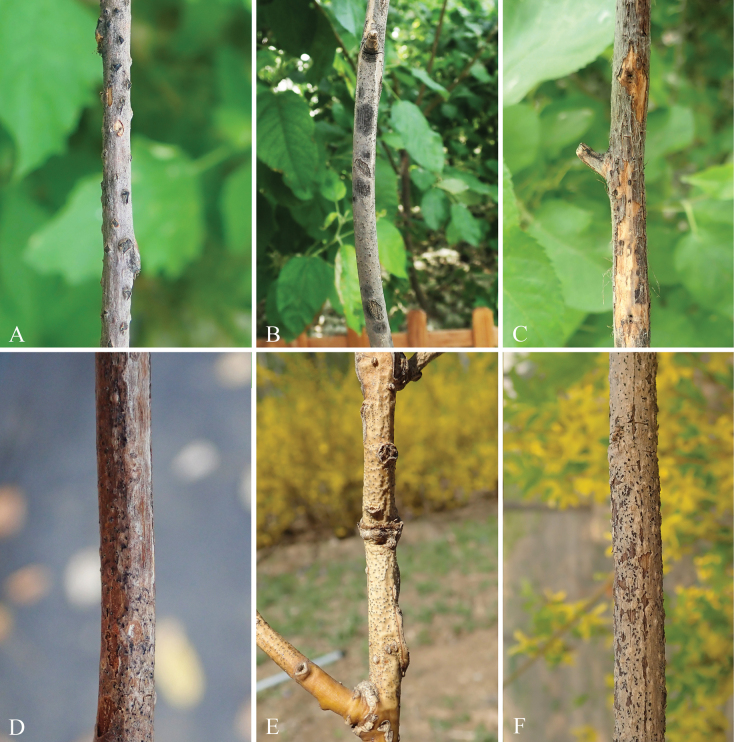
Disease symptoms associated with Botryosphaeriales species collected from Tongzhou District, Beijing, China **A***Xanthocerassorbifolium***B***Fraxinuschinensis***C***Lagerstroemiaindica***D***Sambucuswilliamsii***E***Styphnolobiumjaponicum***F***Forsythiasuspensa*.

A survey on dieback diseases was conducted from March to November 2023 in the Tongzhou District of Beijing, China. A total of thirteen tree species were examined, namely *Acermiyabei*, *A.truncatum*, Cotinuscoggygriavar.cinereus, *Forsythiasuspensa*, *Fraxinuschinensis*, *Ginkgobiloba*, *Lagerstroemiaindica*, *Sambucuswilliamsii*, *Styphnolobiumjaponicum*, *Syringaoblata*, *Syringavulgaris*, *Ulmuspumila*, and *Xanthocerassorbifolium*. Twenty specimens showing typical dieback symptoms (Fig. 1) with typical conidiomata and/or ascomata were collected. All samples were placed in paper bags and transported to the laboratory. Specimens with typical conidiomata pycnidial were selected for isolation. Removing the spore mass from conidiomata and generating single spore colonies or plating superficially sterilized diseased tissue on potato dextrose agar plates (PDA; containing 200 g potatoes, 20 g dextrose, and 20 g agar per liter) and incubating Petri dishes at 25 °C in the dark for 2–3 d. When colonies just formed, they transferred to fresh PDA Petri dishes (Crous et al. 2019). All specimens were deposited at the Museum of Beijing Forestry University (BJFC), and all cultures were preserved at the China Forestry Culture Collection Center (CFCC).

### ﻿Morphological observation

Cultures were incubated on PDA at 25 °C in a 12-h day/night regime (Crous et al. 2019). After 14 days, the colonies were measured, and characteristics based on the color, shape, and sparseness of the aerial mycelium of the pathogen colonies were observed and recorded. Conidiomata were manually sectioned with a double-edged razor blade. Observations were conducted using a Leica DM 2,500 dissecting microscope (Wetzlar, Germany) and a Nikon Eclipse 80i compound microscope, equipped with differential interference contrast (DIC) illumination. Images were captured using a Nis DS-Ri2 camera with the Nikon Nis-Elements F4.30.01 software. Conidial length was measured from the base of the basal cell to the base of the apical appendage, while conidial width was measured at its widest point. A randomized selection of conidia was used for measurement (n = 50).

### ﻿DNA extraction, PCR amplification, and sequencing

Genetic DNA was extracted using the cetyltrime-thylammonium bromide (CTAB) method when the mycelium was well spread on the PDA. DNA samples were stored at -20 °C. The PCR reaction primers (forward and reverse) and amplification conditions are detailed in Table 1. Polymerase chain reaction (PCR) amplification was run on a PTC-200 Thermal Cycler amplifier from Bio-Rad, USA. The PCR amplification systems were all 20 μL, including 10 μL of Mix (Promega), 7 μL of double deionized water, 1 μL each of pre- and post-primers, and 1 μL of DNA template. PCR products were assayed by electrophoresis on 2% agarose gels. Amplified PCR products were sent to a commercial sequencing provider (Tsingke Biotechnology Co. Ltd., Beijing, China).

**Table 1.** Genes used in this study with PCR primers.

Locus	PCR primers	PCR: thermal cycles: (Annealing temp. in bold)	References
ITS	ITS1/ITS4	(95 °C: 30 s, **51 °C**: 30 s, 72 °C: 1 min) × 35 cycles	White et al. 1990
LSU	LROR/LR5	(95 °C: 45 s, **55 °C**: 30 s, 72 °C: 1 min) × 35 cycles	Vilgalys and Hester 1990
*tef1-α*	EF1-728F/EF1-986R	(95 °C: 15 s, **55 °C**: 30 s, 72 °C: 1 min) × 35 cycles	Carbone and Kohn 1999
*tub2*	Bt2a/Bt2b	(95 °C: 30 s, **55 °C**: 30 s, 72 °C: 1 min) × 35 cycles	Glass and Donaldson 1995

### ﻿Phylogenetic analyses

The sequences obtained were assembled using SeqMan v. 7.1.0 software, and reference sequences from related publications (Phillips et al. 2019; Li et al. 2023; Lin et al. 2023a; Wu et al. 2023) were retrieved from the National Center for Biotechnology Information (NCBI; https://www.ncbi.nlm.nih.gov). All sequences generated in this study were submitted to GenBank (Table 2). Sequences were aligned in MAFFT v. 7 at the web server (https://mafft.cbrc.jp/alignment/server/) (Katoh and Standley 2013; Katoh et al. 2019) and further adjustments and editing of the sequences were made with MEGA v. 6 (Tamura et al. 2013). Maximum parsimony (MP), maximum likelihood (ML), and Bayesian inference (BI) were selected to construct phylogenetic trees using PAUP v. 4.0b10, PhyML 3.0, and MrBayes V3.1.2 (Huelsenbeck and Ronquist 2001; Swofford 2003; Silvestro and Michalak 2012). Phylograms were visualized with FigTree v. 1.4.0 (http://tree.bio.ed.ac.uk/software/figtree/) and additional edited with Adobe Illustrator CS v. 5 (Adobe Systems Inc., USA). Maximum-parsimony bootstrap values (MPBP) and maximum-likelihood bootstrap values (MLBP) ≥ 50% and Bayesian posterior probabilities (BYPP) ≥ 0.90 are shown for each tree.

**Table 2.** Isolates of *Aplosporella*, *Dothiorella*, and *Phaeobotryon* used in the molecular analyses in this study. Notes: NA: not applicable, Strains in this study are marked in bold, T: ex-type strains.

Species	Strain	Host	Origin	GenBank accession numbers			
				ITS	*tef1-α*	*tub2*	LSU
*Aplosporellaafricana*	CBS 121777^T^	*Acaciamellifera*	Namibia	KF766196	EU101360	NA	NA
*A.africana*	CBS 1217778^T^	*Acaciamellifera*	Namibia	EU101316	EU101361	NA	NA
*A.artocarpi*	CPC 22791^T^	*Artocarpusheterophyllus*	Thailand	KM006450	KM006481	NA	NA
*A.ginkgonis*	CFCC 52442^T^	*Rhustyphina*	China	MH133916	MH133950	NA	NA
*A.ginkgonis*	CFCC 89661^T^	*Rhustyphina*	China	KM030583	KM030597	NA	NA
***A.ginkgonis***	CFCC 70746	Cotinuscoggygriavar.cinereus	China	PP188498	PP541796	NA	NA
*A.hesperidica*	CBS 732.79^T^	*Citrusaurantium*	Buenos Aires	KX464083	NA	NA	NA
*A.hesperidica*	CBS 208.37	*Citrussinensis*	Zimbabwe	JX681069	NA	NA	NA
*A.javeedii*	CFCC 50054^T^	*Juniperuschinensis*	China	KP208840	KP208846	NA	NA
*A.javeedii*	CFCC 50052	*Gleditsiasinensis*	China	KP208838	KP208844	NA	NA
*A.javeedii*	CFCC 58330	*Populuscanadensis*	China	OQ651161	OQ692921	NA	NA
*A.javeedii*	CFCC 58329	*Populusbeijingensis*	China	OQ651162	OQ692922	NA	NA
*A.javeedii*	CFCC 58412	Populusalbavar.pyramidalis	China	OQ651163	OQ692923	NA	NA
***A.javeedii***	CFCC 70733	*Styphnolobiumjaponicum*	China	PP188499	PP541797	NA	NA
***A.javeedii***	CFCC 70734	*Forsythiasuspensa*	China	PP188500	PP541798	NA	NA
***A.javeedii***	CFCC 70735	*Forsythiasuspensa*	China	PP188501	PP541799	NA	NA
***A.javeedii***	CFCC 70736	*Ulmuspumila*	China	PP188502	PP541800	NA	NA
***A.javeedii***	CFCC 70737	*Acertruncatum*	China	PP188503	PP541801	NA	NA
***A.javeedii***	CFCC 70739	*Sambucuswilliamsii*	China	PP188504	PP541802	NA	NA
***A.javeedii***	CFCC 70740	*Acermiyabei*	China	PP188505	PP541803	NA	NA
***A.javeedii***	CFCC 70741	*Lagerstroemiaindica*	China	PP188506	PP541804	NA	NA
***A.javeedii***	CFCC 70742	*Xanthocerassorbifolium*	China	PP188507	PP541805	NA	NA
***A.javeedii***	CFCC 70744	*Syringavulgaris*	China	PP188508	PP541806	NA	NA
***A.javeedii***	CFCC 70745	*Ulmuspumila*	China	PP188509	PP541807	NA	NA
*A.macropycnidia*	CGMCC 3.17725^T^	*Cerasusyedoensis*	China	KT343648	KX011176	NA	NA
*A.macropycnidia*	CGMCC 3.17726	*Cerasusyedoensis*	China	KT343649	KX011177	NA	NA
*A.papillata*	CBS 121780^T^	*Acaciatortillas*	South Africa	EU101328	EU101373	NA	NA
*A.papillata*	CBS 121781	*Acaciatortillas*	South Africa	EU101329	EU101374	NA	NA
*A.prunicola*	CBS 121167^T^	Prunuspersicavar.nucipersica	South Africa	KF766147	NA	NA	NA
*A.prunicola*	STE-U 6326	Prunuspersicavar.nucipersica	South Africa	EF564375	NA	NA	NA
*A.sophorae*	CPC 29688^T^	*Sophoramicrophylla*	New Zealand North	KY173388	NA	NA	NA
*A.thailandica*	MFLU 16-0615^T^	Dead stems	Thailand	KX423536	KX423537	NA	NA
*A.yalgorensis*	MUCC511^T^	*Acaciacochlearis*	Australia	EF591926	EF591977	NA	NA
*A.yalgorensis*	MUCC512	*Eucalyptusgomphocephala*	Australia	EF591927	EF591978	NA	NA
*A.yanqingensis*	CFCC 58791^T^	*Platycladusorientalis*	China	OQ651164	OQ692924	NA	NA
*A.yanqingensis*	CFCC 58792^T^	*Platycladusorientalis*	China	OQ651165	OQ692925	NA	NA
***A.yanqingensis***	CFCC 70743	*Acertruncatum*	China	PP188510	PP541808	NA	NA
***A.yanqingensis***	CFCC 70738	*Acertruncatum*	China	PP188511	PP541809	NA	NA
*Alanomycesindica*	CBS 134264^T^	Soil	India	HF563622	AB872219	NA	NA
*Dothiorellaalpina*	CGMCC 3-18001^T^	*Platycladusorientalis*	China	KX499645	KX499651	NA	NA
*Do.acacicola*	CBS 141295^T^	*Acaciamearnsii*	Réunion	KX228269	KX228376	NA	NA
*Do.acericola*	KUMCC 18-0137^T^	*Acerpalmatum*	China	MK359449	MK361182	NA	NA
***Do.acericola***	CFCC 70755	*Forsythiasuspensa*	China	PP188520	PP766251	PP566659	NA
***Do.acericola***	CFCC 70760	*Ginkgobiloba*	China	PP188521	PP766252	PP566660	NA
***Do.acericola***	CFCC 70761	*Syringaoblata*	China	PP188522	PP766253	PP566661	NA
*Do.albiziae*	MFLUCC 22-0057^T^	*Albizialebbeck*	Thailand	ON751762	ON799588	ON799590	NA
*Do.alpina*	CFCC 58299^T^	*Populusszechuanica*	China	OQ651166	OQ692932	OQ692926	NA
*Do.americana*	CBS 128309^T^	*Vitis species and Vitisvinifera*	USA: Missouri	HQ288218	HQ288262	HQ288297	NA
*Do.baihuashanensis*	CFCC 58549^T^	*Juniperuschinensis*	China	OQ651167	OQ692933	OQ692927	NA
*Do.baihuashanensis*	CFCC 58788^T^	*Juniperuschinensis*	China	OQ651168	OQ692934	OQ692928	NA
*Do.brevicollis*	CBS 130411 = CMW 36463^T^	*Acaciakarroo*	South Africa	JQ239403	JQ239390	JQ239371	NA
*Do.californica*	CBS 119635	*Laurusnobilis*	Turkey	MT587396	MT592108	MT592579	NA
*Do.californica*	CBS 141587^T^	*Umbellulariacalifornica*	USA	KX357188	KX357211	KX357165	NA
*Do.camelliae*	CMGCC 3.24158^T^	*Camelliaoleifera*	China	OQ190531	OQ241464	OQ275064	NA
*Do.capri-amissi*	CBS 121763 = CMW 25403 = CAMS 1158^T^	*Acaciaerioloba*	South Africa	EU101323	EU101368	KX464850	NA
*Do.capri-amissi*	CBS 121878 = CMW 25404 = CAMS 1159^T^	*Acaciaerioloba*	South Africa	EU101324	EU101369	KX464851	NA
*Do.casuarinae*	CBS 120688 = CMW 4855^T^	*Casuarina* sp.	Australia	DQ846773	DQ875331	DQ875340	NA
*Do.casuarinae*	CBS 120689 = CMW 4856	*Casuarina* sp.	Australia	DQ846772	DQ875332	DQ875339	NA
*Do.casuarinae*	CBS 120690 = CMW 4857	*Casuarina* sp.	Australia	DQ846774	DQ875333	DQ875341	NA
*Do.citricola*	CBS 124728 = ICMP 16827	*Citrussinensis*	New Zealand	EU673322	EU673289	KX464852	NA
*Do.citricola*	CBS 124729 = ICMP 16828^T^	*Citrussinensis*	New Zealand	EU673323	EU673290	KX464853	NA
*Do.citrimurotticola*	BE5 = CGMCC3.20392^T^	*Citrusunshiu*	China	MW880663	MW884166	MW884195	NA
*Do.citrimurotticola*	BE8 = CGMCC3.20394	*Citrusreticulatachen* × *C.sinensis*	China	MW880661	MW884164	MW884193	NA
*Do.diospyricola*	CBS 145972	*Diospyrosmespiliformis*	South Africa	MT587398	MT592110	MT592581	NA
*Do.dulcispinae*	CBS 121764 = CMW 25406 = CAMS 1159	*Acaciamellifera*	Namibia	EU101299	EU101344	KX464854	NA
*Do.dulcispinae*	CBS 130413 = CMW 36460^T^	*Acaciakarroo*	South Africa	JQ239400	JQ239387	JQ239373	NA
*Do.eriobotryae*	CBS 140852^T^	*Eriobotryajaponica*	Spain	KT240287	KT240262	MT592582	NA
*Do.franceschinii*	CBS 147722	*Rhamnusalaternus*	Italy	OP999677	OQ067247	NA	NA
*Do.guttulata*	MFLUCC 17-0242	*Alnusglutinosa*	Italy	KY797637	NA	NA	NA
*Do.heterophyllae*	CMW 46458^T^	*Acaciaheterophylla*	Réunion	MN103794	MH548348	MH548324	NA
***Do.hortiarborum***	CFCC 70756^T^	*Fraxinuschinensis*	China	PP188523	PP723042	PP566662	NA
***Do.hortiarborum***	CFCC 70757	*Fraxinuschinensis*	China	PP188524	PP723043	PP566663	NA
***Do.hortiarborum***	CFCC 70758	*Lagerstroemiaindica*	China	PP188525	PP723044	PP566664	NA
***Do.hortiarborum***	CFCC 70759	*Lagerstroemiaindica*	China	PP188526	PP723045	PP566665	NA
*Do.iberica*	CBS 113188 = DA-1	*Quercussuber*	Spain	AY573198	EU673278	EU673097	NA
*Do.iberica*	CBS 113189 = DE-14	*Quercusilex*	Spain	AY573199	AY573230	KX464855	NA
*Do.iberica*	CBS 115041 = CAP 145^T^	*Quercusilex*	Spain	AY573202	AY573222	EU673096	NA
*Do.irannica*	CBS 124722 = CJA 153 = IRAN 1587C^T^	*Oleaeuropaea*	Iran, Golestan	KC898231	KC898214	KX464856	NA
*Do.koae*	CMW 48017^T^	*Acaciakoa*	Hawaiian Is.	MH447652	MH548338	MH548327	NA
*Do.lampangensis*	MFLUCC 18-0232^T^	Rutaceae	Thailand	MK347758	MK340869	MK412874	NA
*Do.longicollis*	CBS 122066 = CMW 26164	*Terminalia* sp.	Australia	EU144052	EU144067	KX464857	NA
*Do.longicollis*	CBS 122067 = CMW 26165	*Lysiphyllumcunninghamii*	Australia	EU144053	EU144068	KX464858	NA
*Do.longicollis*	CBS 122068 = CMW 26166^T^	*Lysiphyllumcunninghamii*	Australia	EU144054	EU144069	KF766130	NA
*Do.magnoliae*	CFCC51563^T^	*Magnoliagrandiflora*	China	KY111247	KY213686	NA	NA
*Do.mangifericola*	CBS 124727^T^	*Mangiferaindica*	Iran	KC898221	KX464614	NA	NA
*Do.mangifericola*	IRAN 1584C	*Mangiferaindica*	Iran	MT587407	MT592119	NA	NA
*Do.moneti*	WAC 13154 = MUCC 505^T^	*Acaciarostellifera*	Australia	EF591920	EF591971	EF591954	NA
*Do.neclivorem*	DAR 80992^T^	*Vitisvinifera*	Australia	KJ573643	KJ573640	KJ577551	NA
*Do.oblonga*	CBS 121765 = CMW 25407 = CAMS 1162^T^	*Acaciamellifera*	South Africa	EU101300	EU101345	KX464862	NA
*Do.oblonga*	CBS 121766 = CMW 25408 = CAMS 1163	*Acaciamellifera*	South Africa	EU101301	EU101346	KX464863	NA
*Do.obovata*	MFLUCC22-0058^T^	*Pavoniaodorata*	Thailand	ON751763	ON799589	ON799591	NA
*Do.omnivora*	CBS 124717 = CJA 214 = IRAN 1570C	*Juglansregia*	Iran	KC898233	KC898216	KX464865	NA
*Do.omnivora*	CBS 392.80	–	France	KX464133	KX464626	KX464897	NA
*Do.omnivora*	CBS 124716 = CJA 241 = IRAN 1573C	*Juglansregia*	Iran	KC898232	KC898215	KX464864	NA
*Do.omnivora*	CBS 242.51	–	Italy	EU673317	EU673284	EU673105	NA
*Do.omnivora*	CBS 188.87	*Juglansregia*	France	EU673316	EU673283	EU673119	NA
*Do.parva*	CBS 124720 = CJA 27 = IRAN 1579C^T^	*Corylus* sp.	Iran	KC898234	KC898217	KX464866	NA
*Do.parva*	CBS 124721 = CJA 35	*Corylus* sp.	Iran	KX464123	KX464615	KX464867	NA
*Do.parva*	CBS 125580	*Corylusavellana*	Austria	KX464124	KX464616	KX464868	NA
*Do.plurivora*	CBS 124724 = CJA 254 = IRAN 1557C^T^	*Citrus* sp.	Iran	KC898225	KC898208	KX464874	NA
*Do.pretoriensis*	CBS 130404 = CMW 36480^T^	*Acaciakarroo*	South Africa	JQ239405	JQ239392	JQ239376	NA
*Do.prunicola*	CBS 124723 = CAP 187 = IRAN 1541C^T^	*Prunusdulcis*	Portugal	EU673313	EU673280	EU673100	NA
*Do.rhamni*	MFLUCC 14-0902^T^	*Rhamnuscathartica*	South European Russia	MF398893	MF398945	NA	NA
*Do.rosulata*	CBS 121760 = CMW 25389 = CAMS 1444^T^	*Acaciakarroo*	Namibia	KF766227	EU101335	KX464877	NA
*Do.rosulata*	CBS 121761 = CMW 25392 = CAMS 1147	*Acaciamellifera*	South Africa	EU101293	EU101338	KX464878	NA
*Do.rosulata*	CBS 121762 = CMW 25395 = CAMS 1150	*Acaciamellifera*	South Africa	EU101319	EU101364	KX464879	NA
*Do.rosulata*	CBS 500.72	*Medicagosativa*	South Africa	EU673318	EU673285	EU673118	NA
*Do.santali*	WAC 13155 = MUCC 509^T^	*Santalumacuminatum*	Australia	EF591924	EF591975	EF591958	NA
*Do.saprophytica*	MFLUCC 23-0210	–	Thailand	OR527239	OR532455	OR532454	NA
*Do.sarmentorum*	IMI 63581b	*Ulmus* sp.	UK: England	AY573212	AY573235	EU673102	NA
*Do.sempervirentis*	IRAN 1581C = CBS 124719	*Cupressussempervirens*	Iran	KC898237	KC898220	KX464885	NA
*Do.sempervirentis*	IRAN 1583C = CBS 124718 = CJA 264^T^	*Cupressussempervirens*	Iran	KC898236	KC898219	KX464884	NA
*Do.* sp.	CBS 121783 = CMW 25432 = CAMS 1187	*Acaciamearnsii*	South Africa	EU101333	EU101378	KX464859	NA
*Do.* sp.	CBS 121784 = CMW 25430 = CAMS 1185	*Acaciamearnsii*	South Africa	EU101331	EU101376	KX464860	NA
*Do.* sp.	CBS 121785 = CMW 25433 = CAMS 1188	*Acaciamearnsii*	South Africa	EU101334	EU101379	KX464861	NA
*Do.striata*	CBS 124730 = ICMP 16819	*Citrussinensis*	New Zealand	EU673320	EU673287	EU673142	NA
*Do.striata*	CBS 124731 = ICMP 16824^T^	*Citrussinensis*	New Zealand	EU673321	EU673288	EU673143	NA
*Do.styphnolobii*	Cr01^T^	*Styphnolobiumjaponicum*	Crym	MH880849	MK069594	NA	NA
*Do.symphoricarpicola*	CPC 33923^T^	*Symphoricarpos*	Italy	MT587414	MT592126	MT592606	NA
*Do.tectonae*	MFLUCC18-0232^T^	*Tectonagrandis*	Thailand	KM396899	KM409637	KM510357	NA
*Do.thailandica*	CBS 133991 = CPC 21557 = MFLUCC 11-0438	*Dead bamboo culm*	Thailand	JX646796	JX646861	JX646844	NA
*Do.thripsita*	CBS 125445 = BRIP 51876a^T^	*Acaciaharpophylla*	Australia	KJ573642	KJ573639	KJ577550	NA
*Do.ulmacea*	CBS 141414^T^	*Ulmuslaevis*	Germany	MT587415	MT592127	MT592608	NA
*Do.uruguayensis*	CBS 124908 = CMW 26763^T^	*Hexachlamisedulis*	Uruguay	EU080923	EU863180	KX464886	NA
*Do.vidmadera*	CBS 621.74	*Pyruscommunis*	Switzerland	KX464129	KX464621	KX464887	NA
*Do.vidmadera*	CBS 725.79^T^	*Pyrusmalus*	Switzerland	KX464130	KX464622	KX464888	NA
*Do.vinea-gemmae*	DAR 81012^T^	*Vitisvinifera*	Australia	KJ573644	KJ573641	KJ577552	NA
*Do.viticola*	CBS 117009^T^	*Vitisvinifera*	Spain	AY905554	AY905559	EU673104	NA
*Do.westralis*	WA10NO01^T^	*Vitisvinifera*	Australia	HM009376	HM800511	NA	NA
*Do.yunnana*	CGMCC 3-17999^T^	*Camellia* sp.	China	KX499643	KX499649	NA	NA
*Do.yunnana*	CGMCC 3-18000	*Camellia* sp.	China	KX499644	KX499650	NA	NA
*Do.zanthoxyli*	CMGCC 3.24159^T^	*Zanthoxylumbungeanum*	Sichuan	OQ190536	OQ241468	OQ275069	NA
*Neofusicoccumluteum*	CBS 562.92^T^	*Actinidiadeliciosa*	New Zealand	MH862376	KX464690	KX464968	NA
*Neofusicoccumparvum*	CMW 9081^T^	*Populusnigra*	New Zealand	AY236943	AY236888	AY236917	NA
*Phaeobotryonaplosporum*	CFCC 53774	*Syzygiumaromaticum*	China	MN215836	MN205996	NA	MN215871
*P.aplosporum*	CFCC 53775^T^	*Rhustyphina*	China	MN215837	NA	NA	MN215872
*P.aplosporum*	CFCC 53776	*Rhustyphina*	China	MN215838	MN205997	NA	MN215873
*P.aplosporum*	CFCC 58596	*Juglansmandshurica*	China	OQ651169	NA	NA	OQ652540
*P.aplosporum*	CFCC 58784	*Juglansmandshurica*	China	OQ651170	NA	NA	OQ652541
*P.cupressi*	CBS 124700 = IRAN 1455C^T^	*Cupressussempervirens*	Iran	FJ919672	FJ919661	NA	KX464538
*P.cupressi*	CBS 124701 = IRAN 1458C	*Cupressussempervirens*	Iran	FJ919671	FJ919660	NA	KX464539
***P.fraxini***	CFCC 70762^T^	*Fraxinuschinensis*	China	PP188527	PP505782	NA	PP177348
***P.fraxini***	CFCC 70763	*Fraxinuschinensis*	China	PP188528	PP505783	NA	PP177349
*P.juniperi*	JU001 ^T^	*Juniperusformosana*	China	OP941637	OP948218	NA	OP941644
*P.juniperi*	JU005	*Juniperusformosana*	China	OP941638	OP948219	NA	OP941645
*P.mali*	XJAU 2930^T^	*Maluspumila*	China	MW326854	MW509519	NA	MW367101
*P.mali*	XJAU 2772	*Juglansregia*	China	MW326853	MW509520	NA	MW367094
*P.mali*	XJAU 2782	*Malus ‘Royalty*’	China	MW326852	MW509516	NA	MW367092
*P.mali*	XJAU 3094	*Elaeagnusangustifolia*	China	MW326858	MW509517	NA	MW367100
*P.mali*	XJAU 3100	*Rhustyphina*	China	MW326878	MW509518	NA	MW367093
*P.mamane*	CBS 122980 = CPC 12440^T^	*Sophorachrysophylla*	USA	EU673332	EU673298	NA	EU673248
*P.mamane*	CPC 12442	*Sophorachrysophylla*	USA	EU673333	EU673299	NA	DQ377899
*P.negundinis*	CAA 797	*Acernegundo*	Russia	KX061513	KX061507	NA	NA
*P.negundinis*	CAA 798	*Ligustrumvulgare*	Russia	KX061514	KX061508	NA	NA
*P.negundinis*	CAA 799	*Forsythiaintermedia*	Russia	KX061515	KX061509	NA	NA
*P.negundinis*	CPC 33384	*Acernugundo*	Ukraine	MT587542	MT592276	NA	MT587323
*P.negundinis*	CPC 33388	*Dead stem*	Ukraine	MT587543	MT592277	NA	MT587324
*P.negundinis*	CPC 34752	*Acernegundo*	Ukraine	MT587544	MT592278	NA	MT587325
*P.negundinis*	MFLUCC 15-0436^T^	*Acernegundo*	Russia	KU820970	KU853997	NA	NA
*P.platycladi*	CFCC 58799^T^	*Platycladusorientalis*	China	OQ651172	OQ692930	NA	OQ652543
*P.platycladi*	CFCC 58800	*Platycladusorientalis*	China	OQ651173	OQ692931	NA	OQ652544
*P.rhoinum*	CFCC 52449	*Rhustyphina*	China	MH133923	MH133957	NA	MH133940
*P.rhoinum*	CFCC 52450^T^	*Rhustyphina*	China	MH133924	MH133958	NA	MH133941
*P.rhois*	CFCC 89662 = CCTCC AF2014017^T^	*Rhustyphina*	China	KM030584	KM030598	NA	KM030591
*P.rhois*	CFCC 89663 = CCTCC AF2014016	*Rhustyphina*	China	KM030585	KM030599	NA	KM030592
*P.rhois*	CFCC 58679^T^	Populusalbavar.pyramidalis	China	OQ651171	OQ692929	NA	OQ652542
*P.spiraeae*	CFCC 53925^T^	*Spiraeasalicifolia*	China	OM049420	NA	NA	OM0049432
*P.spiraeae*	CFCC 53926	*Spiraeasalicifolia*	China	OM049421	NA	NA	OM0049433
*P.spiraeae*	CFCC 53927	*Spiraeasalicifolia*	China	OM049422	NA	NA	OM0049434
*P.ulmi*	94-13	*Ulmuspumila*	USA	AF243398	NA	NA	NA
*P.ulmi*	CBS 114123 = UPSC 2552	*Ulmusglabra*	Sweden	MT587539	MT592273	NA	MT587320
*P.ulmi*	CBS 138854 = CPC 24264^T^	*Ulmuslaevis*	Germany	MT587540	MT592274	NA	MT587321
*P.ulmi*	CBS 123.30 = ATCC 24443	*Ulmus* sp.	USA	KX464232	KX464766	NA	DQ377861
*P.ulmi*	CBS 174.63	*Ulmusglabra*	Finland	MT587541	MT592275	NA	MT587322
*P.ulmi*	CMH 299	*House dust*	USA	KF800390	NA	NA	NA
*P.ulmi*	PB_11f	*Ulmusglabra*	Poland	MK134682	NA	NA	NA
*Alanphillipsiaaloeicola*	CBS 138896 = CPC 23674^T^	*Aloe* sp.	South Africa	KP004444	MT592027	NA	KP004472

Maximum parsimony analysis was performed using the tree bisection and reconnection (TBR) branch swapping algorithm with a heuristic search option of 1000 random-addition sequences (Swofford 2003). Max trees were set to 5000 branches of zero length, and all parsimonious trees were saved. Other measures calculated were tree length (TL), consistency index (CI), retention index (RI), and rescaled consistency (RC) (Swofford 2003). Maximum likelihood analysis was performed with the GTR GAMMA model of site substitution, including estimation of gamma-distributed rate heterogeneity and a proportion of invariant sites (Guindon et al. 2010). The branch support from MP and ML analysis was evaluated with a bootstrapping (BS) method of 1 000 replicates (Hillis and Bull 1993). The Bayesian inference analysis employing a Markov chain Monte Carlo (MCMC) algorithm was performed with Bayesian posterior probabilities (Rannala and Yang 1996). The model of nucleotide substitution was estimated by MrModeltest v.2.3 (Posada and Crandall 1998), and a weighted Bayesian analysis was considered. Two MCMC chains were run starting from random trees for 1,000,000 generations and stopped when the average standard deviation of split frequencies fell below 0.01; the trees were sampled every 100^th^ generation. The first 25% of trees were discarded as the burn-in phase of each analysis, and the Bayesian posterior probabilities (BPP) were calculated using the remaining 7,500 trees.

## ﻿Result

### ﻿Phylogenetic analysis

The BLAST results indicated that the 23 isolates resided in *Aplosporella*, *Dothiorella*, and *Phaeobotryon* (14 for *Aplosporella*, 7 for *Dothiorella*, and 2 for *Phaeobotryon*). Separate phylogenetic trees for each of the three genera were constructed in this study.

**Figure 2.**
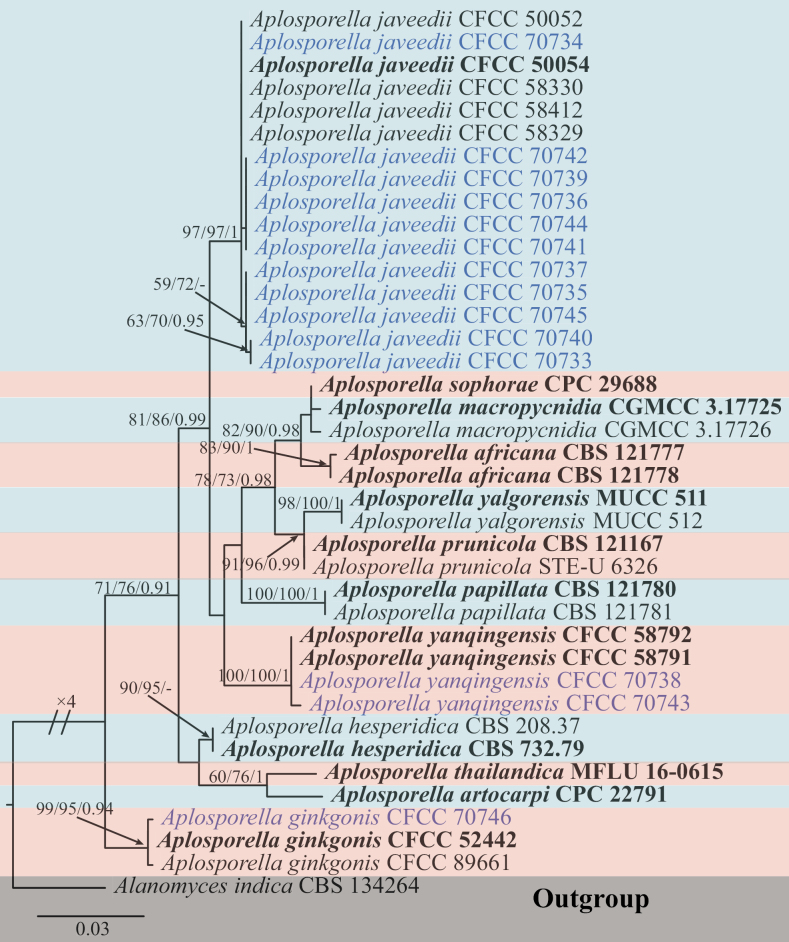
Phylogram generated from RAxML analysis based on ITS with *tef1-α* sequence data of *Aplosporella* isolates. The tree was rooted in *Alanomycesindica* (CBS 134264). The MP, ML (≥ 50%), and BI (≥ 0.9) bootstrap supports are given near the nodes, respectively. Isolates from this study are marked in blue, and ex-type strains are marked in bold.

In *Aplosporella*, the combined ITS and *tef1-α* dataset consists of 944 characters, including alignment gaps (508 for ITS and 436 for *tef1-α*), of which 794 are constant and 60 are variable parsimony uninformative characters. MP analysis with the remaining 90 parsimony-informative characters resulted in one equally parsimonious tree: tree length (TL) = 230; consistency index (CI) = 0.817; retention index (RI) = 0.896; and rescaled consistency index (RC) = 0.732. In ML analysis based on the combined gene dataset, the matrix had 193 distinct alignment patterns. Estimated base frequencies are as follows: A = 0.217607, C = 0.264598, G = 0.259539, T = 0.258256, AC = 2.784746, AG = 2.845183, AT = 1.353935, CG = 1.848853, CT = 4.935430, GT = 1.000000, gamma distribution shape parameter: α = 0.157110, and likelihood value of ln: -2 499.855852. The maximum likelihood (ML) and Bayesian methods (BI) for phylogenetic analyses have the same topology and terminal clades. Fourteen isolates were distributed in *Aplosporella*, aggregated with three known species, *A.javeedii*, *A.yanqingensis*, and *A.ginkgonis*, separately (Fig. 2). The single gene tree for ITS and *tef1-α* of *Aplosporella* is shown in Suppl. material 1.

**Figure 3.**
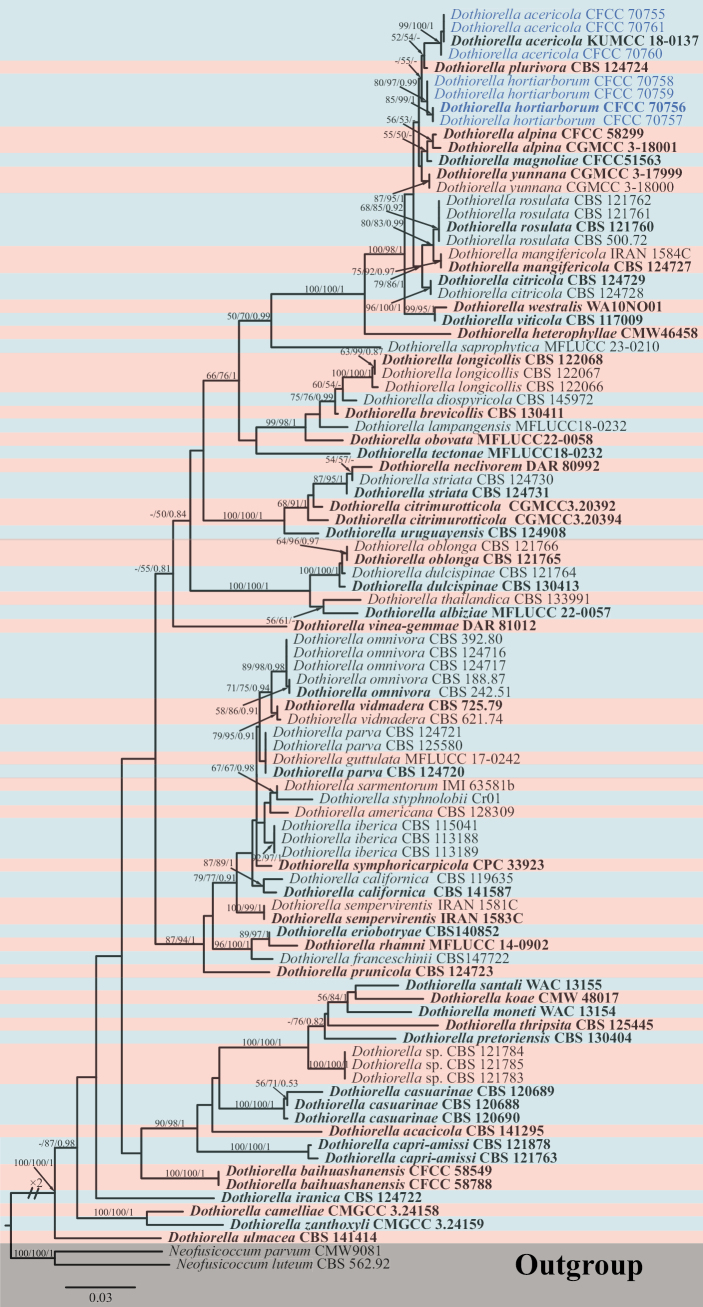
Phylogram generated from RAxML analysis based on ITS, *tef1-α*, and *tub2* sequence data of *Dothiorella* isolates. The tree was rooted in *Neofusicoccumluteum* (CBS 562.92) and *Neofusicoccumparvum* (CMW9081). The MP, ML (≥ 50%), and BI (≥ 0.9) bootstrap supports are given near the nodes, respectively. Isolates from this study are marked in blue, and ex-type strains are marked in bold.

In *Dothiorella*, sequences of the combined ITS, *tef1-α*, and *tub2* were aligned; the dataset consists of 1,319 characters, including alignment gaps (534 for ITS, 369 for *tef1-α*, and 416 for *tub2*), of which 905 are constant and 107 are variable parsimony uninformative characters. MP analysis with the remaining 307 parsimony-informative characters resulted in one equally parsimonious tree: tree length (TL) = 1,282; consistency index (CI) = 0.477; retention index (RI) = 0.824; and rescaled consistency index (RC) = 0.394. In ML analysis based on the combined gene dataset, the matrix had 601 distinct alignment patterns. Estimated base frequencies are as follows: A = 0.206208, C = 0.312741, G = 0.250328, T = 0.230723, AC = 0.833804, AG = 2.174710, AT = 1.041501, CG = 0.791470, CT = 3.735830, GT = 1.000000, gamma distribution shape parameter: α = 0.215045, and likelihood value of ln: -8 567.497788. Three of the seven isolates were of the known species *Dothiorellaacericola*, and the other four isolates formed a separate clade for designation as new species based on phylogenetic analysis (Fig. 3). The single gene tree for ITS, *tef1-α*, and *tub2* of *Dothiorella* is shown in Suppl. material 2.

**Figure 4.**
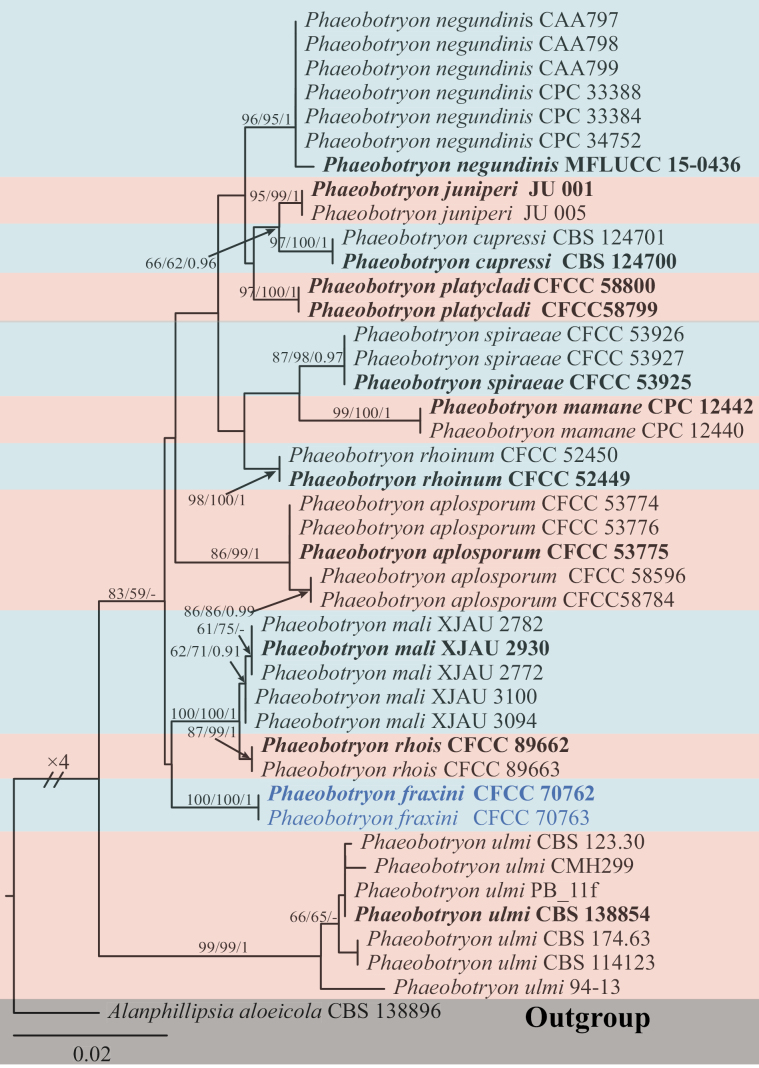
Phylogram generated from RAxML analysis based on ITS, LSU, and *tef1-α* sequence data of *Phaeobotryon* isolates. The tree was rooted in *Alanphillipsiaaloeicola* (CBS 138896). The MP, ML (≥ 50%), and BI (≥ 0.9) bootstrap supports are given near the nodes, respectively. Isolates from this study are marked in blue, and ex-type strains are marked in bold.

In *Phaeobotryon*, the combined ITS, LSU, and *tef1-α* dataset consists of 1,394 characters, including alignment gaps (494 for ITS, 333 for LSU, and 567 for *tef1-α*), of which 1,218 are constant and 56 are variable parsimony uninformative characters. MP analysis with the remaining 120 parsimony-informative characters resulted in one equally parsimonious tree: tree length (TL) = 259; consistency index (CI) = 0.799; retention index (RI) = 0.913; and rescaled consistency index (RC) = 0.730. In ML analysis based on the combined gene dataset, the matrix had 239 distinct alignment patterns. Estimated base frequencies are as follows: A = 0.224820, C = 0.266099, G = 0.277247, T = 0.231833, AC = 0.602998, AG = 2.181745, AT = 0.500445, CG = 0.607508, CT = 4.549533, GT = 1.000000, gamma distribution shape parameter: α = 0.020014, and likelihood value of ln: -3 357.887099. Eight isolates were assigned to *Phaeobotryon*, one isolate aggregated with *P.mali*, and two isolates stood alone, not branching off from known species, representing a new species (Fig. 4). The single gene tree for ITS, LSU, and *tef1-α* of *Phaeobotryon* is shown in Suppl. material 3.

### ﻿Taxonomy

#### ﻿ Aplosporella ginkgonis

Taxon classificationFungiBotryosphaerialesAplosporellaceae

C.M. Tian, Z. Du & K.D. Hyde, Mycosphere 8(2): 1249 (2017)

79F7AB3F-B491-577F-BAD5-3F25F5A70EB6

##### Description.

See Du et al. 2017.

##### Material examined.

China, Beijing City, Tongzhou District, Majuqiao Wetland Park, 39°46'12"N, 116°37'12"E, on the disease branches of Cotinuscoggygriavar.cinereus, 2 May 2023, Y.Y. Wu, BJFC-S1931, living culture CFCC 70746.

##### Notes.

*Aplosporellaginkgonis* was first reported in Gansu Province, China, causing canker and dieback disease in *Ginkgobiloba* and *Morusalba* (Du et al. 2017). Zhu et al. (2018) and Li et al. (2023) discovered the species on *Rhustyphina* and *Zanthoxylumbungeanum*, respectively, extending its host range. In the present study, one isolate (CFCC 70746) was identified as *A.ginkgonis* based on the phylogenetically highly supported clade with 99% MP, 95% ML, and 0.94 BYPP values (Fig. 2) and morphological characteristics. This is the first report of *A.ginkgonis* on Cotinuscoggygriavar.cinereus.

#### ﻿ Aplosporella javeedii

Taxon classificationFungiBotryosphaerialesAplosporellaceae

Jami, Gryzenh., Slippers & M.J. Wingf., Fungal Biology 118(2): 174 (2013)

ADEA9599-379D-57BB-9F13-338D1078DEA4

##### Description.

See Fan et al. 2015.

##### Material examined.

China, Beijing City, Tongzhou District, Hougezhuang Plain Forest, 29°50'24"N, 116°54'00"E, on the dead branches of *Styphnolobiumjaponicum*, 8 April 2023, C.M. Tian, S.J. Li & Y.Y. Wu, BJFC-S1932, living culture CFCC 70733; *ibid*. on the dead branches of *Forsythiasuspensa*, BJFC-S1933, living culture CFCC 70734; *ibid*. on the dead branches of *Forsythiasuspensa*, BJFC-S1934, living culture CFCC 70735; *ibid*. on the dead branches of *Ulmuspumila*, BJFC-S1935, living culture CFCC 70736; China, Beijing City, Tongzhou District, Central Green Forest Park, 39°52'16"N, 116°42'04"E, from branches of *Acertruncatum*, 12 April 2023, C.M. Tian, Y.M. Liang, C. Peng, Y. Hu & Y.Y. Wu, BJFC-S1936, living culture CFCC 70737; China, Beijing City, Tongzhou District, Central Green Forest Park, 39°52'16"N, 116°42'04"E, on the dead branches of *Sambucuswilliamsii*, 19 April 2023, C.M. Tian, C. Peng, R. Yuan, M.W. Zhang & Y.Y. Wu, BJFC-S1937, living culture CFCC 70739; *ibid*. on the dead branches of *Acermiyabei*, BJFC-S1938, living culture CFCC 70740; *ibid*. on the dead branches of *Lagerstroemiaindica*, BJFC-S1939, living culture CFCC 70741; *ibid*. on the dead branches of *Xanthocerassorbifolium*, BJFC-S1940, living culture CFCC 70742; China, Beijing City, Tongzhou District, Majuqiao Wetland Park, 39°46'12"N, 116°37'12"E, from branches of *Syringavulgaris*, 2 May 2023, Y.Y. Wu, BJFC-S1941, living culture CFCC 70744, *ibid*. on the dead branches of *Ulmuspumila*, BJFC-S1942, living culture CFCC 70745.

##### Notes.

*Aplosporellajaveedii* was initially reported on *Celtisafricana* and *Searsialancea* in South Africa (Jami et al. 2014). Fan et al. (2015a) recorded this species in China for the first time, associating it with the canker or dieback disease of five hosts: *Albiziajulibrissin*, *Broussonetiapapyrifera*, *Gleditsiasinensis*, *Juniperuschinensis*, and *Styphnolobiumjaponicum*. *Aplosporellajaveedii* is widespread on host plants of more than 10 families (Fan et al. 2015a; Zhu et al. 2018; Pan et al. 2019; Lin et al. 2023a). In this study, we report new host records for this species, including *Acermiyabei*, *Acertruncatum*, *Forsythiasuspensa*, *Lagerstroemiaindica*, *Sambucuswilliamsii*, *Syringavulgaris*, *Ulmuspumila*, and *Xanthocerassorbifolium*.

#### ﻿ Aplosporella yanqingensis

Taxon classificationFungiBotryosphaerialesAplosporellaceae

L. Lin & X.L. Fan, MycoKeys 97: 9 (2023)

DF3687A9-6028-5DF4-A503-12E5F9D56A35

##### Description.

See Lin et al. 2023a.

##### Material examined.

China, Beijing City, Tongzhou District, Central Green Forest Park, 39°52'16"N, 116°42'04"E, on the dead branches of *Acertruncatum*, 12 April 2023, C.M. Tian, Y.M. Liang, C. Peng, Y. Hu & Y.Y. Wu, BJFC-S1943, living culture CFCC 70743; *ibid*. BJFC-S1944, living culture CFCC 70738.

##### Notes.

*Aplosporellayanqingensis* was first discovered on the branches of *Platycladusorientalis* in Beijing (Lin et al. 2023a). In this study, the two isolates (CFCC 70738 and CFCC 70743) from *Acertruncatum* formed a clade with 100% MP, 100% ML, and 1.00 BYPP values in the multi-locus phylogenetic tree with *A.yanqingensis* (Fig. 2). Compared with the description of Lin et al. (2023a), this study has shorter conidia and thinner conidiogenous cells (11.0–16.5 × 6.0–9.0 µm vs. 16.0–21.5 × 6.0–9.5 µm and 5.0–20.5 × 1.0–2.0 µm vs. 6.0–13.5 × 2.0–3.0 µm). Thus, these isolates were identified as *A.yanqingensis*, and herewith we are providing a new host record for *A.yanqingensis*, *Acertruncatum*.

#### ﻿ Dothiorella acericola

Taxon classificationFungiBotryosphaerialesAplosporellaceae

Phookamsak, Tennakoon & K.D. Hyde, Fungal Diversity 95: 78 (2019)

633FCCBD-BE3E-5432-BAB9-EB0FD2E5D053

##### Description.

See Pan et al. 2021.

##### Material examined.

China, Beijing City, Tongzhou District, Hougezhuang Plain Forest, 29°50'24"N, 116°54'00"E, on the dead branches of *Forsythiasuspensa*, 8 April 2023, C.M. Tian, S.J. Li & Y.Y. Wu, BJFC-S1948, living culture CFCC 70755; China, Beijing City, Tongzhou District, Majuqiao Wetland Park, 39°46'12"N, 116°37'12"E, on the dead branches of *Ginkgobiloba*, 2 May 2023, Y.Y. Wu, BJFC-S1949, living culture CFCC 70760; *ibid*. on the dead branches of *Syringaoblata*, BJFC-S1950, living culture CFCC 70761.

##### Notes.

Based on phylogenetic analyses (Fig. 3), three isolates in this study clustered with *Dothiorellaacericola* and formed a clade with 99% MP, 100% ML, and 1.00 BYPP values. *Dothiorellaacericola* is reported to be associated with the canker disease of *Acerpalmatum* in China (Phookamsak et al. 2019). Pan et al. (2021, 2023) found that *Do.acericola* infests *Ziziphusjujuba* and *Koelreuteriapaniculata* branches. The fungus was also recorded on dead branches of *Euonymusjaponicus* (Lin et al. 2023b). This is the first discovery of this fungus in the host families Oleaceae and Ginkgoaceae.

#### ﻿ Dothiorella hortiarborum

Taxon classificationFungiBotryosphaerialesAplosporellaceae

Y.Y. Wu & C.M. Tian sp. nov.

3066142A-8652-51A7-B99F-99B1DAEB48E5

851826

Fig. 5

##### Etymology.

“Hort” means “garden,” and “arbor” means “tree” in Latin. Collected from *Fraxinuschinensis* and *Lagerstroemiaindica*, both of which are landscaping and greening trees.

##### Holotype.

China, Beijing City, Tongzhou District, Central Green Forest Park, 39°52'16"N, 116°42'04"E, on the dead branches of *Fraxinuschinensis*, 19 April 2023, C.M. Tian, C. Peng, R. Yuan, M.W. Zhang & Y.Y. Wu (holotype BJFC-S1951, ex-type cultures CFCC 70756).

##### Description.

***Sexual morph***: Not observed. ***Asexual morph*: *Conidiomata*** pycnidial, scattered to aggregated, immersed to semi-immersed in bark, globose to subglobose, dark gray to black, unilocular, 260–450 μm diam. ***Disc*** black, ovoid, 310–330 μm diam. ***Ostioles*** single, light gray, circular, central, papillate, 30–45 μm diam. ***Locules*** single, black, oval, 100–380 μm, ***Conidiophores*** reduced to conidiogenous cells. ***Conidiogenous cells***: hyaline, smooth, thin-walled, holoblastic, cylindrical to subcylindrical, 4.5–11.0 × 2.0–4.0 μm (av. ± S.D.= 6.8 ± 1.3 × 2.9 ± 0.5 µm). ***Conidia*** initially hyaline, then producing light yellow pigmentation, uneven surface, thick-walled, dark brown when matrues, 1-septate, constricted at the septum, smooth, ovoid with a broadly rounded apex, truncate base. 10.0–19.0 × 6.0–11.0 μm (av. ± S.D.= 14.9 ± 2.6 × 8.1 ± 1.0 µm).

**Figure 5.**
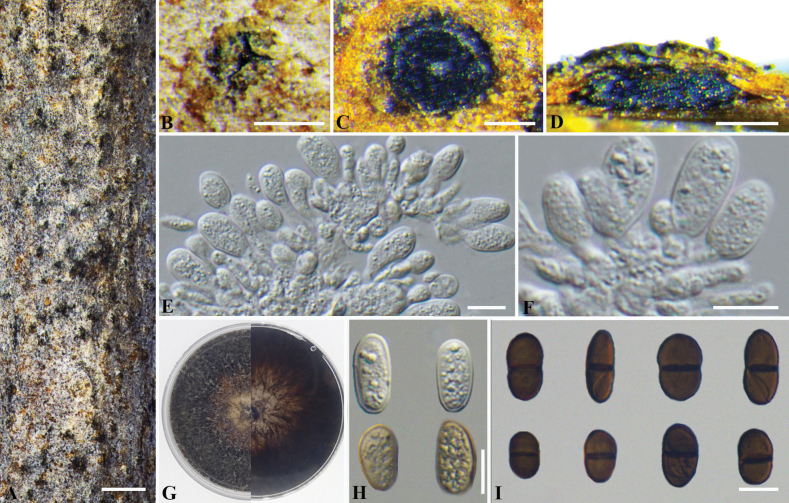
*Dothiorellahortiarborum* (BJFC-S1951) **A, B** habit of conidiomata on branch **C** transverse section of conidioma **D** longitudinal section through conidioma **E, F** conidiogenous cells and conidia **G** top (left) and bottom (right) sides of colonies on potato dextrose agar (PDA) **H, I** conidia. Scale bars: 1000 μm (**A**); 200 μm (**B–D**); 10 μm (**E–F, H–I**).

##### Culture characters.

Colonies on PDA with aerial mycelium gray-green, thick and dense, fluffly, margin with undulate and irregular, reverse with inky blue pigment accumulation, reaching 60 mm diam in 7 days at 25 °C.

##### Other material examined.

China, Beijing City, Tongzhou District, Central Green Forest Park, 39°52'16"N, 116°42'04"E, on the dead branches of *Fraxinuschinensis*, 19 April 2023, C.M. Tian, C. Peng, R. Yuan, M.W. Zhang & Y.Y. Wu, BJFC-S2366, living culture CFCC 70757; China, Beijing City, Tongzhou District, Central Green Forest Park, 39°52'16"N, 116°42'04"E, on the dead branches of *Lagerstroemiaindica*, 19 April 2023, C.M. Tian, C. Peng, R. Yuan, M.W. Zhang & Y.Y. Wu, BJFC-S1952, living culture CFCC 70758; *ibid*. BJFC-S2367, living culture CFCC 70759.

##### Notes.

*Dothiorellahortiarborum* formed an independent clade with 87% MP, 97% ML, and 0.99 BYPP values and is distinct from *Do.acericola* and *Do.plurivora* in the multi-locus analyses (Fig. 3). Morphologically, *Do.hortiarborum* can be distinguished from *Do.acericola* by shorter conidia (Phookamsak et al. 2019) and *Do.plurivora* by smaller conidia (10.0–19.0 × 6.0–11.0 μm vs. 22.3–22.7 × 10.8–11.2 μm) (Abdollahzadeh et al. 2014). Additionally, *Do.hortiarborum* differs from *Do.acericola* in *tef1-α* (five bp difference from 170 characters, with 97.1% similarity, including no gaps) sequences, and *Do.plurivora* in *tef1-α* (one bp difference from 254 characters, with 99.6% similarity, including one gap), *tub2* (three bp difference from 370 characters, with 99.2% similarity, including one gap) sequences.

#### ﻿ Phaeobotryon fraxini

Taxon classificationFungiBotryosphaerialesAplosporellaceae

Y.Y. Wu & C.M. Tian sp. nov.

98414295-A95A-59D7-96BE-C58B9C92917E

851827

Fig. 6

##### Etymology.

Named after the host, *Fraxinuschinensis*.

##### Holotype.

China, Beijing City, Tongzhou District, Central Green Forest Park, 39°52'16"N, 116°42'04"E, on the dead branches of *Fraxinuschinensis*, 19 April 2023, C.M. Tian, C. Peng, R. Yuan, M.W. Zhang & Y.Y. Wu (holotype BJFC-S1953, ex-type cultures CFCC 70762).

##### Description.

***Sexual morph***: Not observed. ***Asexual morph*: *Conidiomata*** pycnidial, scattered, occasionally aggregated, superficial or immersed, globose, dark brown to black, unilocular, 200–360 μm diam. ***Disc*** inconspicuous. ***Ostioles*** single, brown or black, circular, central, papillate, 40–85 μm diam. ***Locules*** single, globose, 100–170 μm, ***Conidiophores*** reduced to conidiogenous cells. ***Conidiogenous cells*** hyaline, smooth, thin-walled, holoblastic, cylindrical, formed from the cells lining the inner walls of the locules, 7.0–14.0 × 1.0–5.0 μm (av. ± S.D.= 10.6 ± 2.0 × 3.1 ± 0.8 µm). ***Conidia*** initially hyaline, smooth, thin-walled, then gradually producing light yellow pigment, becoming yellow or light brown, occasionally with bubbles, mature with 1-septate, brownish yellow to dark brown, oblong, obtuse, rounded at both ends, 13.0–20.0 × 7.0–10.0 μm (av. ± S.D.= 17.6 ± 1.3 × 8.7 ± 0.7 µm).

**Figure 6.**
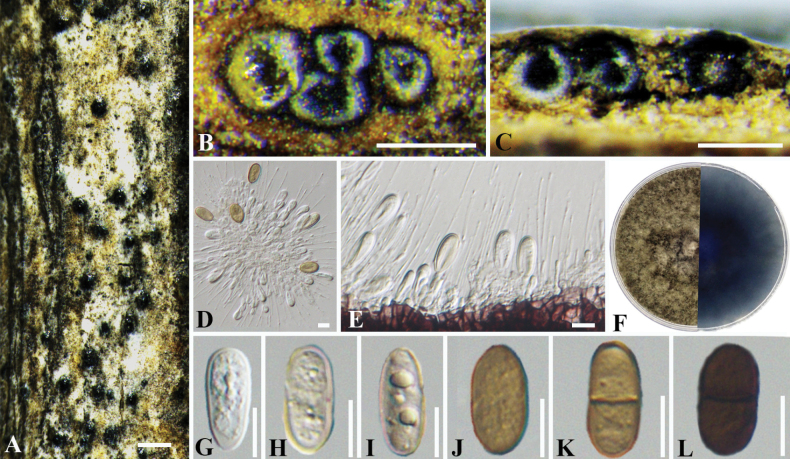
*Phaeobotryonfraxini* (BJFC-S1953) **A** habit of conidiomata on branch **B** transverse section of conidioma **C** longitudinal section through conidioma **D, E** conidiogenous cells and conidia **F** top (left) and bottom (right) sides of colonies on potato dextrose agar (PDA) **G**-**L** conidia. Scale bars: 500 μm (**A**); 200 μm (**B, C**); 10 μm (**D, E, G–L**).

##### Culture characters.

Colonies on PDA with aerial gray-white mycelium, thick and dark black at the edge, thin and paler in color in the center, fluffly, entire margin, reverse with black pigment accumulation, reaching 60 mm diam in 7 days at 25 °C.

##### Other material examined.

China, Beijing City, Tongzhou District, Central Green Forest Park, 39°52'16"N, 116°42'04"E, on the dead branches of *Fraxinuschinensis*, 19 April 2023, C.M. Tian, C. Peng, R. Yuan, M.W. Zhang & Y.Y. Wu, BJFC-S2368, living culture CFCC 70763.

##### Notes.

Based on multi-locus phylogenetic analysis, the two isolates cluster separately in a high-supported clade with 100% MP, 100% ML, and 1.00 BYPP value (Fig. 4). In the phylogenetic analysis, *Phaeobotryonfraxini* showed a close relationship to *P.mali* and *P.rhois.* These three species could be distinguished based on ITS, *tef1-α*, and LSU loci from *P.mali* by nineteen bp (6/465 in ITS; 10/184 in *tef1-α*; 3/559 in LSU) and *P.rhois* by twenty-two bp (7/465 in ITS; 12/184 in *tef1-α*; 3/559 in LSU). Moreover, *P.fraxini* differs from *P.mali* and *P.rhois* in having smaller conidia (13.0–20.0 × 7.0–10.0 µm vs. 22.0–31.5 × 12–16.5 µm for *P.mali* and 20–25 × 10–12 µm for *P.rhois*) (Fan et al. 2015b; Jia et al. 2023) (Table 3). Therefore, *P.fraxini* is introduced as a novel species.

**Table 3.** Comparison of species in *Phaeobotryon*.

Species	Host	Location	Conidial size	Septation	Reference
*Phaeobotryonaplosporum*	*Rhustyphina*	China	17–19 × 5.5–7	aseptate	Pan et al. 2019
*P.mali*	*Maluspumila*	China	22.0–31.5 × 12–16.5	1-septate	Jia et al. 2023
*P.cupressi*	*Cupressussempervirens*	Iran	24.1–25 × 12.2–12.5	1(–2)-septate	Abdollahzadeh et al. 2009
*P.fraxini*	*Fraxinuschinensis*	China	13–20 × 7–10	1-septate	This study
*P.juniperi*	*Juniperusformosana*	China	24.5–27.5 × 12.0–13.5	1-septate	Peng et al. 2023
*P.mamane*	*Sophorachrysophylla*	USA	35–38 × 14–15	1(–2)-septate	Phillips et al. 2008
*P.negundinis*	*Acernegundo*	Russia	16–24.5 × 7.9–11.5	aseptate	Daranagama et al. 2016
*P.platycladi*	*Platycladusorientalis*	China	23.0–31.0 × 9.5–12.5	aseptate or 1-septate	Lin et al. 2023a
*P.rhoinum*	*Rhustyphina*	China	19–21 × 7.5–9	1-septate	Zhu et al. 2018
*P.rhois*	*Rhustyphina*	China	20–25 × 10–12	1-septate	Fan et al. 2015b
*P.spiraeae*	*Spiraeasalicifolia*	China	23.5–28.5 × 8.5–13.5	aseptate	Jin and Karunarathna 2021
*P.ulmi*	*Ulmuslaevis*	Germany	28.5–32.5 × 16.5–18.5	aseptate or 1-septate	Zhang et al. 2021

## ﻿Discussion

In this paper, 23 Botryosphaeriales isolates were identified as six species based on multi-locus phylogenetic analyses. These species included two new species, namely *Dothiorellahortiarborum* and *Phaeobotryonfraxini*, and four new hosts: *Aplosporellaginkgonis* on Cotinuscoggygriavar.cinereus; *A.javeedii* on *Acermiyabei*; *Acertruncatum*; *Forsythiasuspensa*; *Lagerstroemiaindica*; *Sambucuswilliamsii*; *Syringavulgaris*; *Ulmuspumila*; *Xanthocerassorbifolium*; *A.yanqingensis* on *Acertruncatum*; and *Do.acericola* on *Forsythiasuspensa*; *Ginkgobiloba*; and *Syringaoblata*. The six fungal species identified in this study involve a total of 13 different hosts, which elucidates the wide range of hosts of Botryospaeriales.

*Aplosporella* is the type genus of Aplosporellaceae (Slippers et al. 2013). The distinctive morphological feature of *Aplosporella* species is that both ascospores and conidia are aseptately hyaline to pigmented (Slippers et al. 2013; Phillips et al. 2019). In this study, a total of three new host record species of the genus were identified, including *A.ginkgonis*, *A.javeedii*, and *A.yanqingensis*. *Aplosporellajaveedii* has the highest isolation rate and the widest host range, involving five orders of host plants, including Dipsacales, Fabales, Lamiales, Myrtales, and Rosales. Currently, this species is mainly found in warm temperate and tropical regions (Fan et al. 2015a; Zhu et al. 2018), and further exploration is needed to determine whether the geographic range of *A.javeedii* is related to climate.

*Dothiorella* was considered a synonym of *Diplodia* based on a broad morphological concept (Crous and Palm 1999). Phillips et al. (2005) compared the morphological characteristics again and found that the conidia of *Dothiorella* were brown, with 1-septate in early development, and the conidia still adhered to the conidiogenous cells. In contrast, the conidia of *Diplodia* become black and septate after being excreted from the conidiomata. Crous et al. (2006) confirmed these morphological differences. Therefore, *Dothiorella* is regarded as an independent genus in the Botryosphaeriaceae. In this study, the conidia of *Do.hortiarborum* are transparent and aseptate when attached to conidiogenous cells. After being released by the conidiomata, the conidia bear yellowish pigment or become brown with a 1-septate. In recent years, many new species of *Dothiorella* have been published with conidial morphology similar to *Do.hortiarborum* (Li et al. 2023; Lin et al. 2023a; Wu et al. 2023). These suggest that the morphological characteristics of *Dothiorella* are not always stable. Thus, it is not accurate to rely solely on the morphology of conidia for *Dothiorella*; combining phylogenetic analysis and the size of conidia of neighboring species is necessary. *Dothiorella* species have been reported on more than 20 host plants in China (https://fungi.ars.usda.gov/). This study has expanded its host range in Oleaceae plants (*Do.acericola* in *Forsythiasuspensa*, *Ginkgobiloba* and *S.oblata*, and *Do.hortiarborum* in *Fraxinuschinensis*).

Currently, many *Dothiorella* species have been recorded from *Fraxinus*, distributed mainly in regions such as Europe and North America (Table 4). In this study, a new species, *Do.hortiarborum*, from *F.chinensis*, was introduced in China. However, based on morphological and DNA sequence data, *Do.hortiarborum* shows significant differences from other species in *Fraxinus*. Phylogenetic analysis showed that *Do.hortiarborum* belongs to a different lineage from *Do.omnivora*, *Do.* sp., and *Do.vidmadera* (Fig. 3), while distinguishing them based on the size of conidia and the number of septates (Table 4). *Do.concaviuscula*, *Do.fraxini*, and *Do.fraxinicola* were not available for sequence information due to their earlier publication; however, *Do.hortiarborum* can also be easily distinguished from them based on their documented conidia size. In addition, *Do.lagerstroemiae* and *Do.hortiarborum* were both isolated from *Lagersiroemiaalba*, but its conidia were significantly smaller than *Do.hortiarborum* (8.3–10 × 3.5–4 µm vs. 10.0–19.0 × 6.0–11.0 μm).

**Table 4.** Comparison of species from *Fraxinus* in *Dothiorella*.

Specise	Host	Location	Conidial size	Septation	Reference
*Dothiorellaconcaviuscula*	*Fraxinusviridis*	USA	4–6 × 2.5–3	no description	Jepson 1896
*Do.fraxini*	*Fraxinus* sp.	Belgium	26–30 × 12	1-septate	Saccardo 1892
*Do.fraxinicola*	*Fraxinus* sp.	USA	18–30 × 6–7	no description	Ellis and Everhart 1895
*Do.hortiarborum*	*Fraxinuschinensis*	China	10.0–19.0 × 6.0–11.0	1-septate	This study
*Do.omnivora*	*Fraxinusexcelsior*	Bosnia	19.3–25.5 × 7.5–10.6	1-septate	Linaldeddu et al. 2016
*Do.* sp.	*Fraxinusexcelsior*	Bosnia, Herzegovina	11–14 × 6–8	2–4-septate	Zlatković et al. 2016
*Do.vidmadera*	*Fraxinusornus*	Australia	21.2–21.9 × 9.6–9.8	1-septate	Pitt et al. 2013

*Phaeobotryon* species have more overlapping morphological characters, with 1(–2) septate or aseptate conidia and similar pigmentation variations. For example, *P.cupressi* and *P.juniperi* have overlapping sizes of conidia (24.1–25 × 12.2–12.5 μm vs. 24.5–27.5 × 12.0–13.5 μm), *P.rhoinum* and *P.rhois* are derived from the same host and geographic origin, and the conidia have 1-septate (Table 3). So, morphology combined with phylogenetics to further clarify the affinities between species is essential. Furthermore, Phaeobotryon species were reported on a variety of hosts and considered to be potential or opportunistic pathogens (Weiland et al. 2016; Zhu et al. 2020; Ilyukhin and Ellouze 2023; Jia et al. 2023). In this study, *P.fraxini* was isolated only from dead *Fraxinuschinensis*; more extensive specimen collection was needed to confirm its distribution characteristics and pathogenicity.

Although Botryosphaeriales recorded many fungi on Index Fungorum (https://www.indexfungorum.org/), only some species are now recognized. Mainly due to the early records of many species, the lack of model specimens, or the low quality of specimens, it is difficult to obtain strains and DNA data. Therefore, more detailed sampling is needed to revise the classification system of related taxa in Botryosphaeriales.

## Supplementary Material

XML Treatment for Aplosporella ginkgonis

XML Treatment for Aplosporella javeedii

XML Treatment for Aplosporella yanqingensis

XML Treatment for Dothiorella acericola

XML Treatment for Dothiorella hortiarborum

XML Treatment for Phaeobotryon fraxini

## ﻿Additional information

### Author ORCIDs

Yingying Wu https://orcid.org/0009-0007-5095-2738

Rong Yuan https://orcid.org/0009-0006-5597-7531

Chengming Tian https://orcid.org/0000-0002-3352-7664

### Data availability

All of the data that support the findings of this study are available in the main text or Supplementary Information.

## Supplementary materials

Supplementary material 1
Aplosporella
Data type pdf https://binary.pensoft.net/file/1080711This dataset is made available under the Open Database License (http://opendatacommons.org/licenses/odbl/1.0/). The Open Database License (ODbL) is a license agreement intended to allow users to freely share, modify, and use this Dataset while maintaining this same freedom for others, provided that the original source and author(s) are credited.Yingying Wu, Cheng Peng, Rong Yuan, Mingwei Zhang, Yang Hu, Chengming Tian

Supplementary material 2
Dothiorella
Data type pdf https://binary.pensoft.net/file/1080712This dataset is made available under the Open Database License (http://opendatacommons.org/licenses/odbl/1.0/). The Open Database License (ODbL) is a license agreement intended to allow users to freely share, modify, and use this Dataset while maintaining this same freedom for others, provided that the original source and author(s) are credited.Yingying Wu, Cheng Peng, Rong Yuan, Mingwei Zhang, Yang Hu, Chengming Tian

Supplementary material 3
Phaeobotryon
Data type pdf https://binary.pensoft.net/file/1080713This dataset is made available under the Open Database License (http://opendatacommons.org/licenses/odbl/1.0/). The Open Database License (ODbL) is a license agreement intended to allow users to freely share, modify, and use this Dataset while maintaining this same freedom for others, provided that the original source and author(s) are credited.Yingying Wu, Cheng Peng, Rong Yuan, Mingwei Zhang, Yang Hu, Chengming Tian
