# Supplementary material for: ﻿New species and records of Botryosphaeriales (Dothideomycetes) associated with tree dieback in Beijing, China
Source: MycoKeys. 2024 Jun 27;106:225–50. doi: 10.3897/mycokeys.106.122890 (PMC11224674; doi:10.3897/mycokeys.106.122890)
Supplement: Supplementary material 2 — Dothiorella [file mycokeys-106-225-s002.pdf]

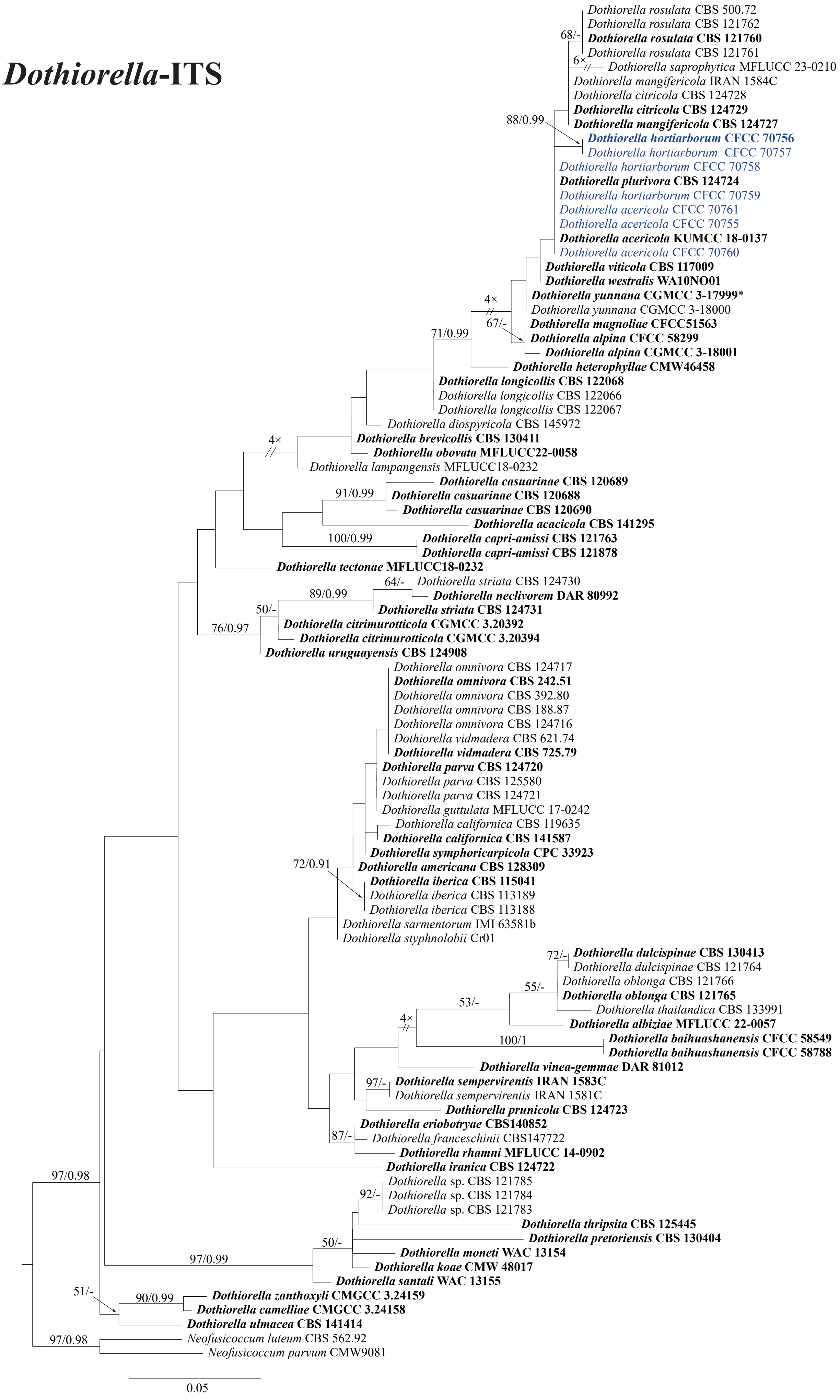

Figure S2-1. Phylogram generated from RAxML analysis based on ITS sequence data of *Dothiorella* isolates. The ML (≥ 50%) and BI (≥ 0.9) bootstrap supports are given near the nodes, respectively.

Dothiorella-tef1-α

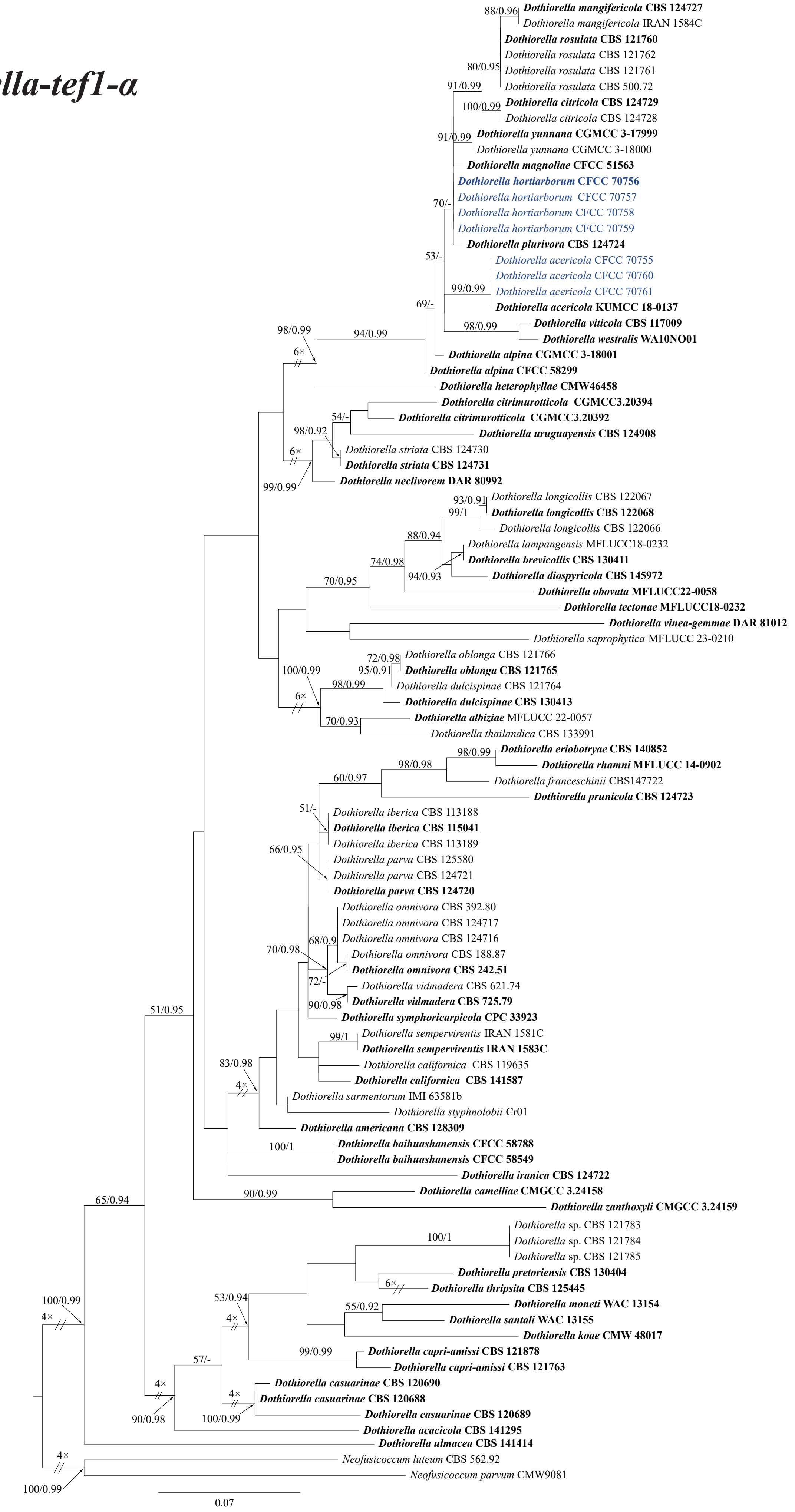

Figure S2-2. Phylogram generated from RAxML analysis based on *tef1-α* sequence data of *Dothiorella* isolates. The ML (≥ 50%) and BI (≥ 0.9) bootstrap supports are given near the nodes, respectively.

Dothiorella-tub2

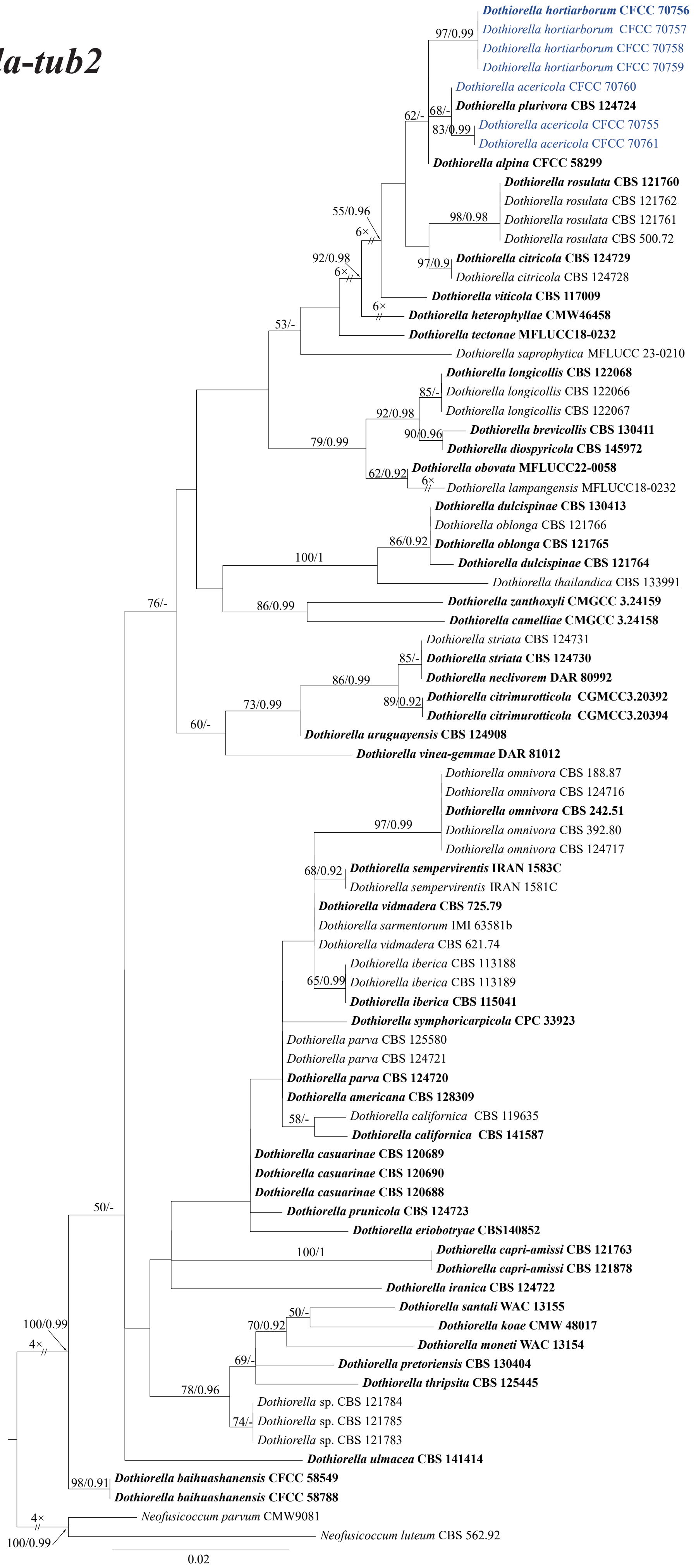

Figure S2-3. Phylogram generated from RAxML analysis based on *tub2* sequence data of *Dothiorella* isolates. The ML (≥ 50%) and BI (≥ 0.9) bootstrap supports are given near the nodes, respectively.
