# Supplementary material for: ﻿New species and records of Botryosphaeriales (Dothideomycetes) associated with tree dieback in Beijing, China
Source: MycoKeys. 2024 Jun 27;106:225–50. doi: 10.3897/mycokeys.106.122890 (PMC11224674; doi:10.3897/mycokeys.106.122890)
Supplement: Supplementary material 3 — Phaeobotryon [file mycokeys-106-225-s003.pdf]

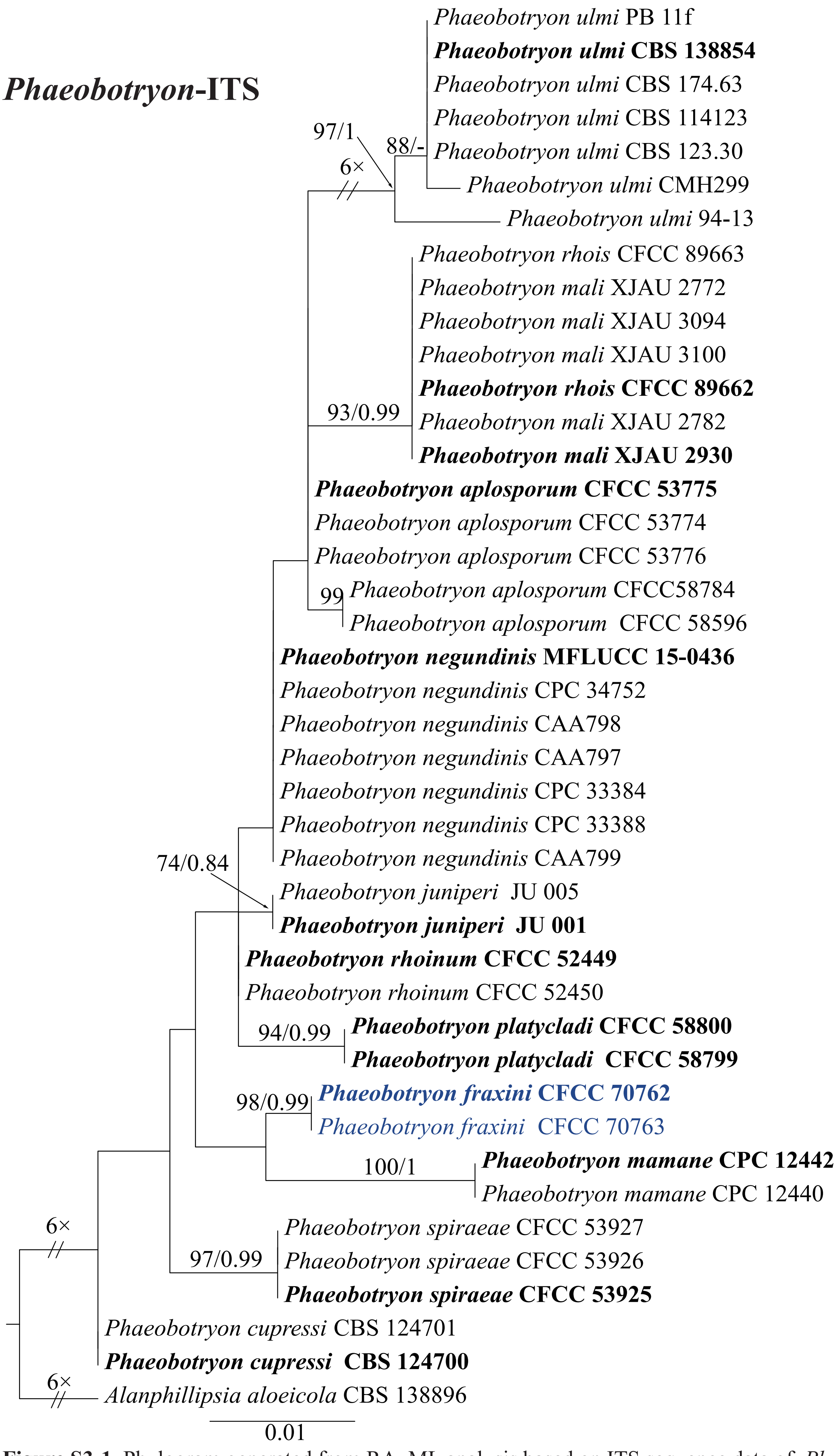


**Figure S3-1.** Phylogram generated from RAxML analysis based on ITS sequence data of *Phaeobotryon* isolates. The ML (≥ 50%) and BI (≥ 0.9) bootstrap supports are given near the nodes, respectively.

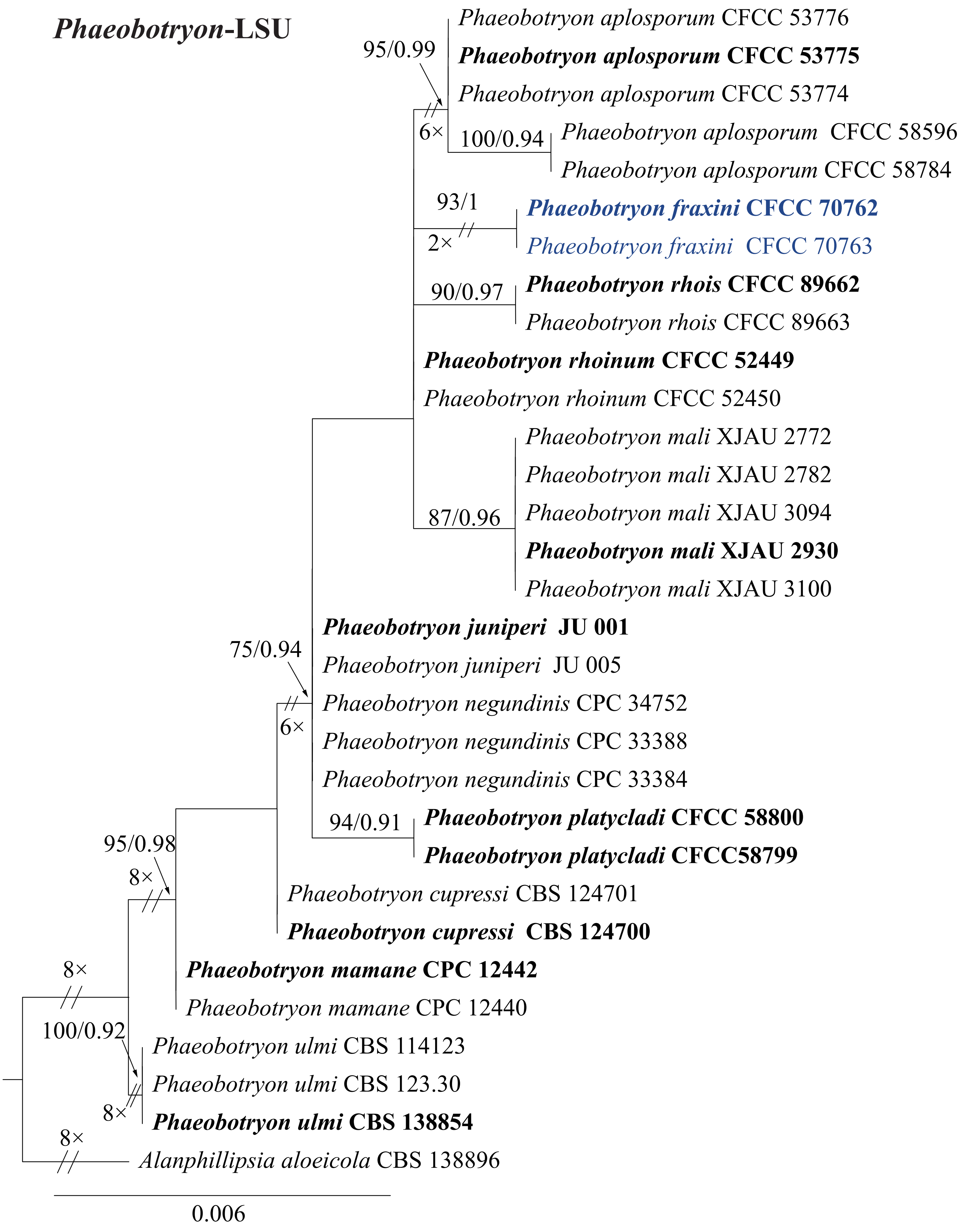


**Figure S3-2.** Phylogram generated from RAxML analysis based on LSU sequence data of *Phaeobotryon* isolates. The ML (≥ 50%) and BI (≥ 0.9) bootstrap supports are given near the nodes, respectively.

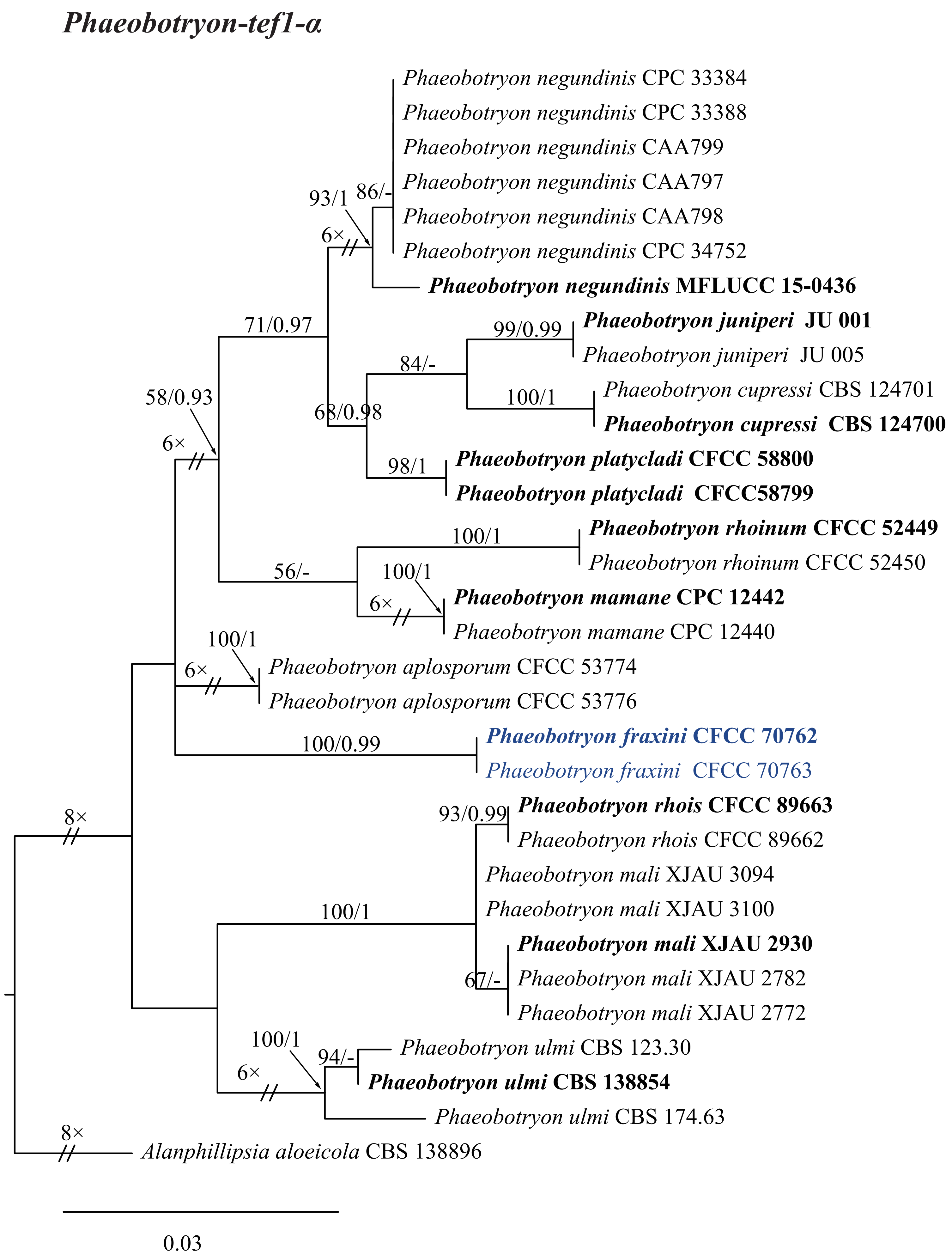


**Figure S3-3.** Phylogram generated from RAxML analysis based on *tef1-α* sequence data of *Phaeobotryon* isolates. The ML (≥ 50%) and BI (≥ 0.9) bootstrap supports are given near the nodes, respectively.
